# Circulating TP73‐AS1 and CRNDE serve as diagnostic and prognostic biomarkers for non‐small cell lung cancer

**DOI:** 10.1002/cam4.5013

**Published:** 2022-07-24

**Authors:** Rong‐Xia Yuan, Chun‐Hua Dai, Ping Chen, Meng‐Jia Lv, Yang Shu, Zhi‐Peng Wang, Ya‐Ping Xu, Jian Li

**Affiliations:** ^1^ Department of Pulmonary Medicine Affiliated Hospital of Jiangsu University Zhenjiang China; ^2^ Department of Respiratory Disease Yancheng Third People's Hospital Yancheng China; ^3^ Department of Radiotherapy Affiliated Hospital of Jiangsu University Zhenjiang China; ^4^ Center of Experimental Medicine Affiliated Hospital of Jiangsu University Zhenjiang China

**Keywords:** circulating lncRNAs, CRNDE, non‐small cell lung cancer, TP73‐AS1

## Abstract

**Background:**

Circulating long noncoding RNAs (lncRNAs) are considered a new class of biomarkers for the diagnosis and prognosis of various malignancies. We aimed to identify circulating lncRNAs as biomarkers for the diagnosis and prognosis of non‐small cell lung cancer (NSCLC).

**Methods:**

The expression of 14 candidate lncRNAs was measured in matched cancer and ipsilateral normal lung tissues of 20 patients with NSCLC using quantitative reverse‐transcription PCR. In plasma samples from training and testing sets, significantly and aberrantly expressed lncRNAs, TA73‐AS1 and CRNDE, were further analyzed. Receiver operating characteristic (ROC) curves were constructed, and the areas under the ROC curves (AUC) were obtained to assess diagnostic performance. The Kaplan–Meier survival analysis was used to assess the impact of plasma TA73‐AS1 and CRNDE expression on tumor‐free survival (TFS) of patients with NSCLC. The effect of TP73‐AS1 expression on NSCLC cells was investigated in vitro.

**Results:**

AUC values of plasma TA73‐AS1 and CRNDE were 0.822 and 0.815 in the training set and 0.843 and 0.804 in the testing set, respectively, to distinguish NSCLC from healthy controls. The combination of plasma TP73‐AS1, CRNDE, and two classical tumor markers, carcinoembryonic antigen (CEA) and cytokeratin 19 fragment (CYFRA21‐1), showed excellent diagnostic performance for NSCLC (AUC =0.927 in the training set; AUC = 0.925 in the testing set). Furthermore, the high expression of the two plasma lncRNAs correlated with worse TFS in patients with NSCLC. In vitro cell model studies revealed that TP73‐AS1 overexpression facilitated NSCLC cell survival, invasion, and migration.

**Conclusion:**

Circulating TP73‐AS1 and CRNDE could be potential biomarkers for the diagnosis and prognostic prediction of NSCLC.

## Graphical abstract

Circulating TP73‐AS1 and CRNDE could serve as biomarkers for diagnosis of NSCLC. Circulating TP73‐AS1 and CRNDE could serve as biomarkers for prognostic prediction of NSCLC.

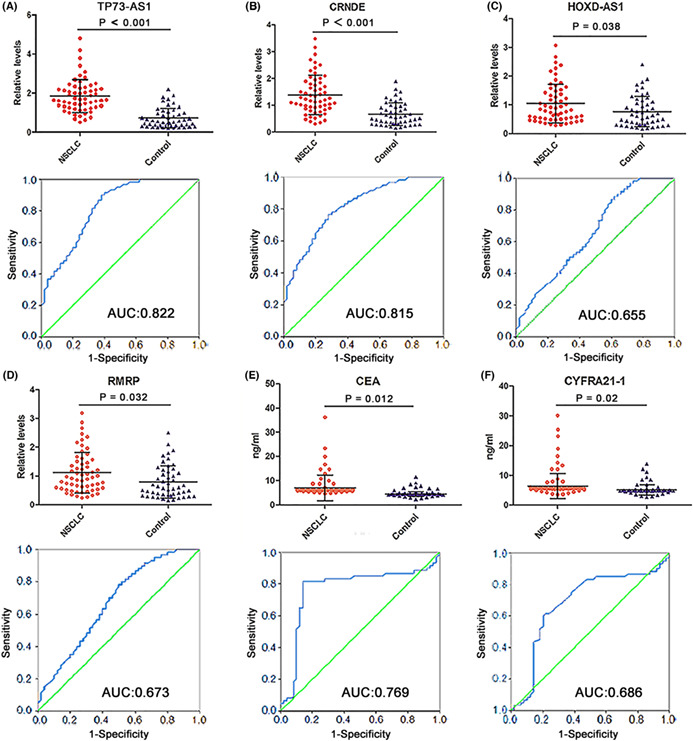

## 1 BACKGROUND

Lung cancer is still the top cause of malignancy‐related mortality worldwide, with the most comment histological type, non‐small cell lung cancer (NSCLC), accounting for nearly 85% of all cases.^1^ Late diagnosis is a major barrier to improving lung cancer prognosis, with an average 5‐year survival rate of roughly 15% for NSCLC owing to diagnosis at advanced stage and delay treatment.^2^ It is crucial from a clinical perspective to pursue effective means for the early detection of NSCLC. Additionally, a high recurrence rate is one of the main causes underlying the poor prognosis of NSCLC.^3^ Currently, few available methods offer a prognostic estimate of patients with NSCLC, which is markedly valuable in selecting effective treatments for these patients.^4^

Long noncoding RNAs (lncRNAs) are a class of noncoding RNAs (ncRNAs) more than 200 nucleotides in length exhibiting limited or no protein‐coding capability.^5^, ^6^ Although the biological effect of lncRNAs needs to be comprehensively clarified, lncRNAs were shown to be involved in oncobiology, including cell growth, apoptosis, invasion, and metastasis.^5^, ^6^, ^7^ The aberrant expression of lncRNAs has been documented in several tumors, including NSCLC, exhibiting oncogenic or tumor‐suppressor properties.^6^, ^7^ Previous studies have shown that patients with NSCLC display a unique lncRNA signature related to NSCLC development, progression, and metastasis.^8^, ^9^, ^10^, ^11^, ^12^ Given their high levels and stability, circulating lncRNAs can be potentially employed as non‐invasive biomarkers to provide information regarding cancer diagnosis and the therapeutic efficacy, such as chemotherapy and targeted therapies.^6^, ^7^ Thus, lncRNAs as biomarkers have been widely investigated in tumor diagnosis and prognostic prediction. However, to our knowledge, few studies have investigated circulating lncRNAs for early diagnosis and prognostic prediction of NSCLC.^13^, ^14^

In the present study, we selected 14 candidate lncRNAs closely related to NSCLC based on previous reports demonstrating that lncRNAs are aberrantly expressed in NSCLC tissues (Table S1). We determined the expression of the 14 lncRNAs in matched cancer and ipsilateral normal lung tissues of 20 patients with NSCLC using quantitative reverse‐transcription PCR (qRT‐PCR). Subsequently, TP73‐AS1 and CRNDE were selected as candidate circulating biomarkers of NSCLC for validation in two cohorts of plasma samples by qRT‐PCR. The diagnostic efficiency of the two lncRNAs was compared with that of the currently used classic tumor markers, carcinoembryonic antigen (CEA) and cytokeratin 19 fragment (CYFRA21‐1). The correlation between the two lncRNAs and tumor‐free survival (TFS) of patients with NSCLC was examined to determine their potential for prognostic prediction. In addition, we assessed the effect of TP73‐AS1 expression on NSCLC cell viability, migration, and invasion in vitro.

## 2 METHODS

### 2.1 Patients and study design

In this study, we analyzed 438 blood samples and 40 tissue samples obtained from patients with stage I‐IIIA NSCLC, healthy controls, and patients with benign lung disease (BLD) at the Affiliated Hospital of Jiangsu University, China, between January 2017 and December 2019. All patients with NSCLC received tumor resection, with respective blood specimens collected before surgery. All patient diagnoses were determined based on histopathological evidence of NSCLC from tissue samples harvested during surgery. NSCLC tumors were staged based on the 7th lung cancer TNM classification and staging system of the International Association for the Study of Lung Cancer (IASLC). All patients did not receive radiotherapy or chemotherapy before surgery.

**FIGURE 1:**
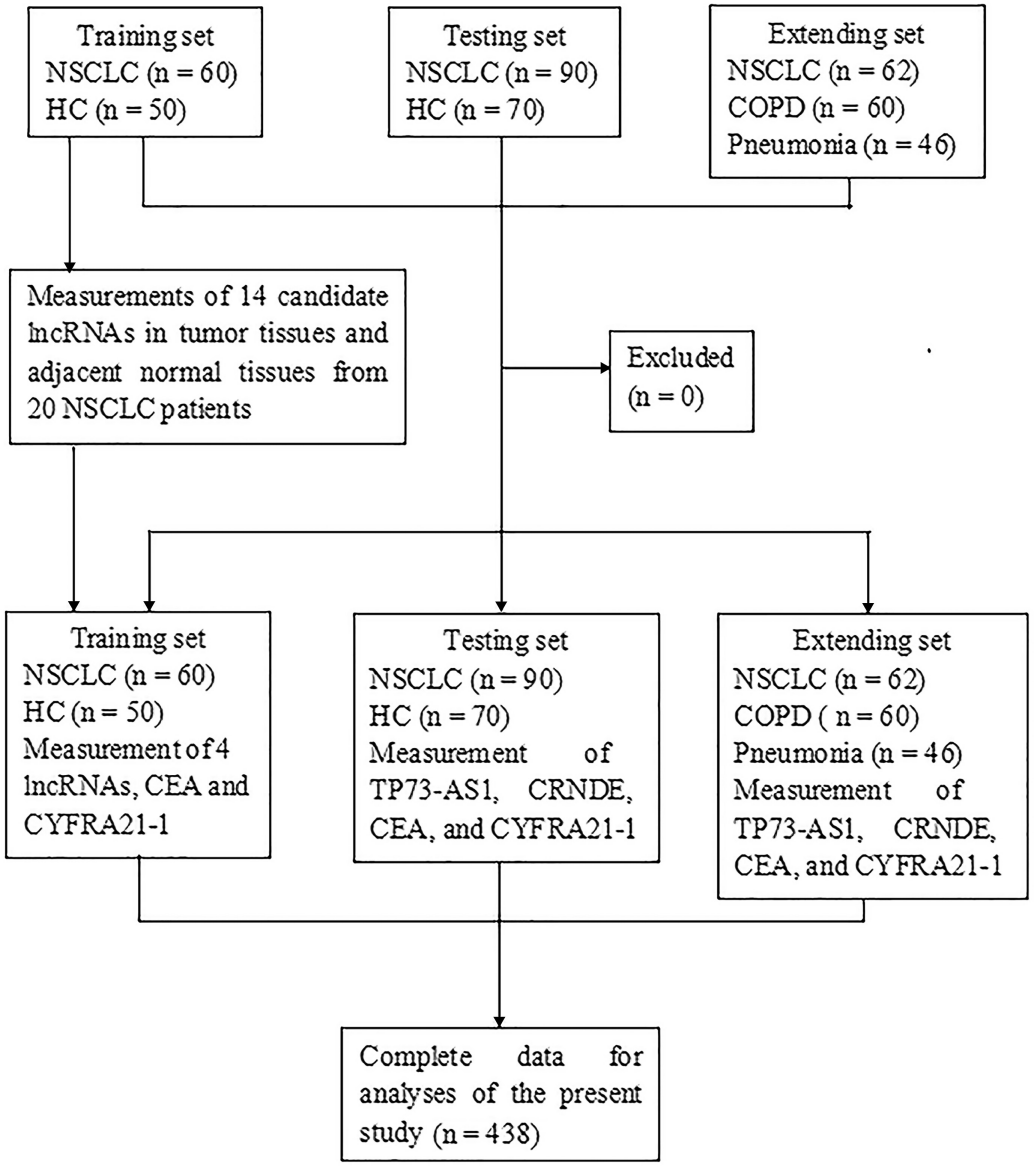
Flow diagram of the study participants. CEA, carcinoembryonic antigen; COPD, chronic obstructive pulmonary disease; CYFRA21‐1, cytokeratin 19 fragment; HC, healthy control; NSCLC, non‐small cell lung cancer.

Figure 1 presents a patient flow diagram explaining the flow of patients and healthy controls throughout the study. During the first phase of this study, 14 candidate lncRNAs, which were reported to be dysregulated in NSCLC (Table S1) in previous studies, were analyzed by qRT‐PCR assay in matched tumor and ipsilateral normal lung tissues obtained from 20 patients with NSCLC, and only lncRNAs with a mean fold‐change ≥2 and *p* < 0.05 were selected for subsequent analysis in the training set. Based on the initial screening results, four lncRNAs (TP73‐AS1, CRNDE, HOXD‐AS1, and RMRP) demonstrated markedly abnormal expression (Table S2). For the training set, we performed quantitative expression analysis of the four lncRNAs in plasma specimens from 60 patients with NSCLC and 50 healthy subjects as controls and compared them with the NSCLC markers CEA, CYFRA21‐1, and squamous cell carcinoma antigen (SCC‐A). We confirmed the significantly elevated levels of TP73‐AS1 and CRNDE in the plasma of patients with NSCLC compared to healthy controls and observed their superior potential to serum CEA, CYFRA21‐1, and SCC‐A in distinguishing NSCLC from healthy controls, whereas plasma HOXD‐AS1 and RMRP were inferior to serum CEA and Cyfra21‐1 in diagnosing NSCLC. Subsequently, we augmented the sample size to 160 plasma specimens from 90 patients with NSCLC and 70 healthy controls in the testing set and validated the high expression of plasma TP73‐AS1 and CRNDE by qRT‐PCR, as well as their diagnostic performance for NSCLC. Finally, we extended the study to patients with BLD (60 with chronic obstructive pulmonary disease [COPD] and 46 with pneumonia) as controls (extending set). The diagnostic performance of each biomarker and their combined use was tested using analyses of receiver operating characteristic (ROC) curves.

In the training and testing sets, 128 patients with stage IB–IIIA NSCLC after surgery underwent 3–5 cycles of adjuvant chemotherapy with a cisplatin‐containing regimen. The patients were followed up for tumor recurrence at specific time intervals. The Kaplan–Meier curve analysis was applied to assess the correlation between plasma TP73‐AS1 and CRNDE levels and TFS in patients with NSCLC. All participants gave written consent, indicating their willingness to donate blood and/or tissue samples for the study. This study was approved by the Ethics Review Board of the Affiliated Hospital of Jiangsu University, China (No. JDFY‐2015029) and was carried out in accordance with the principles of the Declaration of Helsinki.

### 2.2 Specimen collection

Herein, up to 5 ml peripheral blood specimens were obtained from each subject. These blood specimens were collected into EDTA‐containing tubes and subjected to two‐step centrifugation (2000*g* for 15 min at 4°C and 3000*g* for 20 min at 4°C) to eliminate cell sediments and prevent cellular nucleic acid contamination. All plasma specimens were stored at −80°C until further analysis. The time from specimen collection to storage at −80°C was ˂2 h.

### 2.3 RNA extraction and qRT‐PCR assay

Total RNA was isolated from 400 μl plasma or tissue samples using TRI 20 L reagent (Invitrogen) following the manufacturer instructions. The RNA concentration was measured using a NanoDrop spectrophotometer. Reverse transcription of the lncRNA was conducted by the PrimeScript™ RT reagent kit (Takara Bio), according to the manufacturer guidelines. Primers for the 14 lncRNAs were synthesized by Ribo Co., Ltd. Primer sequences for the 14 lncRNAs are presented in Table S3. qRT‐PCR assay was performed with SYBR Premix ExTaq (Takara Bio) in a 25‐μl reaction system containing 12 μl of SYBR Premix ExTaqu, 1 μl of ROX Reference Dye, 1 μl of forward primer (10 μM), 1.5 μl of reverse primer (10 μM), 2.5 μl of cDNA product, and 7 μl of RNase‐free dH_2_O. The solution was incubated at 95°C for 30 s, followed by 60°C for 1 min. All experiments were carried out in triplicate. U6 snRNA was employed as a reference gene. The relative expression level of each lncRNA was calculated relative to that of the U6 snRNA using the 2^−∆Ct^ method.

### 2.4 Cells and culture condition

The human NSCLC cell lines A549 and Calu‐1 were obtained from the Cell Bank of the Chinese Academy Sciences (Shanghai, China) and were periodically authenticated (FuHeng Cell Center). The cells were incubated in RPMI 1640 medium with 10% fetal calf serum (ScienCell) and antibiotics (100 IU/ml penicillin and 100 mg/ml streptomycin) at 37°C, 95% humidity, and 5% CO_2_.

### 2.5 Plasmid construction and transfection

For TP73‐AS1 overexpression, the primary precursor sequence of TP73‐AS1 was amplified by PCR and cloned into a lentiviral plasmid PLenti‐DEST lentivector (Thermo Fisher Scientific). A549 and Calu‐1 cells were cultured overnight in 12‐well plates at a density of 4 **×** 10^6^ cells/well. The cells were washed twice with phosphate‐buffered saline. The two cell lines were transfected with the TP73‐AS1 lentivirus vector or the empty vector in the presence of ViraPower™ Packaging Kit Mix (Thermo Fisher Scientific) and 8 mg/ml polybrene.

### 2.6 siRNA transfection

Two siRNAs targeting TP73‐AS1 (siTP73‐AS1‐1^#^ and siTP73‐AS1‐2^#^) and a non‐target siRNA (siNC) were synthesized by Ribo Co., Ltd. A549 and Calu‐1 cells were incubated in 6‐well plates for 24 h and then transfected with siRNA against TP73‐AS1 or siNC (100 nM) using Lipofectamine 2000 (Ribo Co., Lt.) following the manufacturer guideline, as previously described.^15^ The efficiency of TP73‐AS1 knockdown was verified by qRT‐PCR.

### 2.7 CCK‐8 assay

After transfection, equal numbers of A549 and Calu‐1 cells were incubated in 96‐well plates (2 **×** 10^3^ cells/well) for 24 h. Cell viability was detected by cell counting kit‐8 (CCK‐8) assay (Beyotime) following the manufacturer guidelines as described previously.^15^ Optical density at 490 nm was detected using a Benchmark Plus TM microplate spectrometer (BioRad).

### 2.8 Colony formation assay

A colony formation assay was conducted as previously reported.^15^, ^16^ Briefly, after transfection with TP73‐AS1 lentiviral lentivector or siRNAs targeting TP73‐AS1, cells were incubated in 6‐well plates (5 **×** 10^3^ cells/well) at 37°C with 5% CO_2_ for 14 days to form colonies. Subsequently, colonies were stained with 0.1% crystal violet, then counted using an inverted microscope.

### 2.9 Cell apoptosis assay

The percent of cell apoptosis was measured by flow cytometry and annexin V‐FITC/PI staining, according to the manufacturer protocol, as previously reported.^16^, ^17^

### 2.10 Wound healing assay

The wound healing assay was carried out as previously reported.^16^ Briefly, transfected cells were incubated and grown to confluence in 6‐well plates (1 **×** 10^6^ cells/well). A small wound area was generated by scratching the cell monolayer with a sterile 1‐ml pipette tip in confluent cultured cells. Then, cells were incubated at 37°C for the indicated time point. The widths of the wound area were imaged using an inverted microscope to assess cell migration at indicated time points after scratching, and the relative width of the wound area was measured by ImageJ software (National Institutes of Health, Bethesda, MD).

### 2.11 Cell invasion assay

For the invasive assay, transwell chambers with 8‐μm pore membranes (Corning, USA) were applied in accordance with the manufacturer instructions, as previously described.^16^ Cells (5 × 10^4^ cells/well) were cultured in the upper chamber in 200 μl serum‐free DMEM, with 600 μl DMEM containing 2.5% fetal calf serum added to each lower chamber. After culturing at 37°C for 24 h, cells invading and adhering to the lower membrane surface were fixed with 4% paraformaldehyde and stained with 0.1% crystal violet for 30 min. Cells were photographed using a light microscope, and invaded cells were counted in five random fields.

### 2.12 Western blot assay

After transfections with vectors or siRNAs, cells were harvested and washed twice with BPS, and then harvested in RIPA buffer (Beyotime Biotechnology). Protein lysates were prepared and analyzed by standard western blot assay as previously reported.^17^

### 2.13 Statistical analyses

All statistical analyses were conducted using SPSS software (version 20.0; IBM Corp.) or GraphPad Prism 5 (GraphPad Software Inc.). Numerical data are showed as mean ± standard deviation (SD). Baseline characteristic comparisons between NSCLC patients and BLD patients, or healthy controls were conducted using the Mann–Whitney test or Kruskal‐Wallis test and the Pearson chi‐square test or Fisher's exact test. Differences of plasma lncRNA levels between NSCLC patients and healthy controls were compared using Student's *t*‐test or the Mann–Whitney test. ROC curves were established, and the area under the ROC curves (AUCs) was analyzed to assess the diagnostic performance of selected biomarkers for NSCLC. Risk scores were assigned to each NSCLC patient according to a linear combination of the blood values of two lncRNAs and two tumor markers, weighted based on the regression coefficient. The diagnostic marker panels were constructed using stepwise logistic regression analysis according to the measured results of training and testing sets. The prediction probability of NSCLC diagnosis from healthy controls was used as an index to establish the ROC curve. AUC obtained was applied as an index to assess the diagnostic efficiency of the marker panels.

The length of TFS was defined as the time from the date of surgery to the date of tumor recurrence or the last follow‐up date. Kaplan–Meier survival curve analyses and the log‐rank test were used to compare the TFS of NSCLC patients with different plasma lncRNA expression levels. Univariate and multivariate Cox regression analyses were carried out to identify independent predictive factors of TFS. *p*‐values <0.05 were considered significant.

## 3 RESULTS

### 3.1 Baseline characteristics of the study population

A total of 438 participants, including 212 patients with NSCLC, 120 healthy controls, 60 patients with COPD, and 46 patients with pneumonia, were included in this study consisting of training, testing, and extension sets (Figure 1). Baseline characteristics of all participants are summarized in Table 1 and Table S4, and no marked differences in the distributions of age, gender, and smoking history were observed between patients with NSCLC and healthy controls in training and testing sets. However, due to restrictions in the sampling of patients with COPD and pneumonia, obvious differences were noted in age and smoking history among the three groups in the extended set (Table S4).

**TABLE 1:** Characteristics of subjects in the training and testing sets

	Training set			Testing set		
	NSCLC	Healthy control	*p* value	NSCLC	Healthy control	*p* value
	(*N* = 60)	(*n* = 50)		(*N* = 90)	(*n* = 70)	
Age (years)						
Mean ± SD	65.7 ± 11.2	61.4 ± 8.5	0.168	66.2 ± 8.9	61.9 ± 10.5	0.153
Gender						
Female	32 (53%)	28 (56%)	0.426	46 (51%)	38 (54%)	0.637
Male	28 (47%)	22 (44%)		44 (49%)	32 (46%)	
Smoking status						
Nonsmoker	24 (40%)	22 (44%)	0.655	42 (47%)	28 (40%)	0.425
Smoker	36 (60%)	28 (56%)		48 (53%)	42 (60%)	
Histology						
Adenocarcinoma	34 (57%)	NA		51 (57%)	NA	
SCC	26 (43%)	NA		39 (43%)	NA	
TNM stage						
I	19 (32%)	NA		31 (35%)	NA	
II	26 (43%)	NA		38 (42%)	NA	
IIIA	15 (25%)	NA		21 (23%)	NA	

Abbreviations: NA, not assessed; SCC, squamous cell carcinoma; TNM, tumor node metastasis.

### 3.2 Selection and analyses of NSCLC‐related lncRNAs in cancer tissue specimens

During the initial selection of candidate lncRNAs, 14 lncRNAs found to be aberrantly expressed in NSCLC tissues and playing an key role in carcinogenesis (Table S1) were analyzed by qRT‐PCR assay in matched 20 NSCLC tissues and ipsilateral normal lung tissues. Overall, 4 of 14 lncRNAs, including TP73‐AS1, CRNDE, HOXD‐AS1, and RMRP, showed markedly high expression in NSCLC tissues compared to normal lung tissues (mean change fold ≥2, *p* < 0.001; Table S2). Accordingly, these four lncRNAs were identified as candidate lncRNA biomarkers in subsequent studies.

### 3.3 Analyses of candidate plasma lncRNAs expressions and diagnostic performance for NSCLC in the training set

**FIGURE 2:**
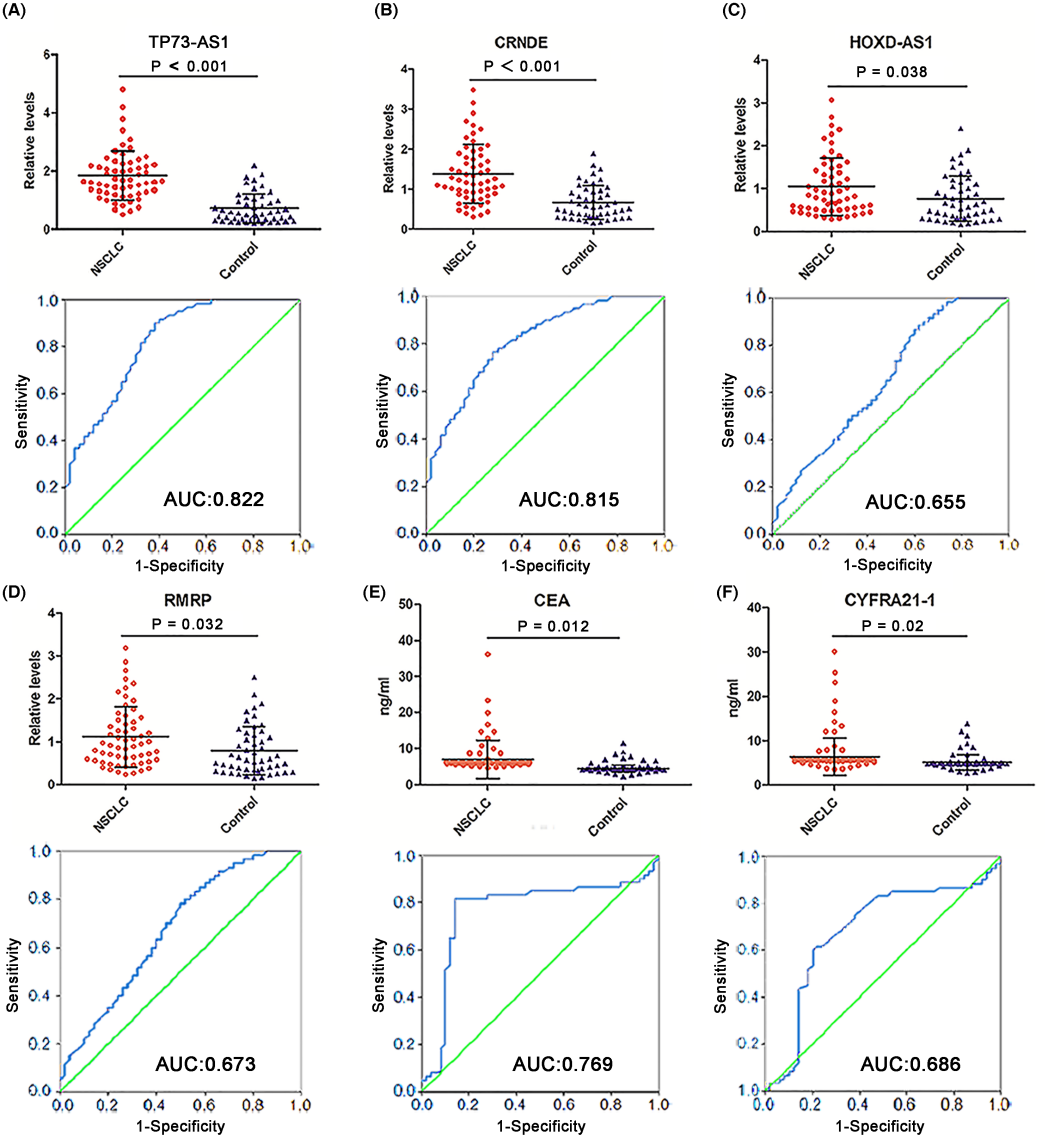
Expression levels and diagnostic efficiency of four plasma lncRNAs and two serum classic tumor markers in the training set. Expression levels of (A) TP73‐AS1, (B) CRNDE, (C) HOXD‐AS1, and (D) RMRP are significantly elevated in the plasma of NSCLC patients compared with healthy controls, as measured by qRT‐PCR. (E) CEA and (F) CYFRA21‐1 levels are markedly elevated in the serum of NSCLC patients compared with healthy controls (upper). The ROC curves and AUCs of TP73‐AS1 (A), CRNDE (B), HOXD‐AS1 (C), RMRP (D), CEA (E), and CYFRA21‐1 (F) in discerning NSCLC patients from healthy controls (lower). AUCs, areas under the ROC curves; CEA, carcinoembryonic antigen; CYFRA21‐1, cytokeratin 19 fragment; lncRNAs, long noncoding RNAs; NSCLC, non‐small cell lung cancer; qRT‐PCR, quantitative reverse‐transcription PCR; ROC, receiver operating characteristic.

To verify the differential expression of the four selected lncRNAs in plasma samples, we tested their relative levels in the training set, which included 60 patients with NSCLC and 50 healthy controls. Concurrently, we measured serum levels of CEA, CYFRA21‐2, and SCC‐A. As shown in Figure 2A,B, plasma TP73‐AS1 and CRNDE levels were dramatically elevated in patients with NSCLC compared to healthy controls (both *p* < 0.001). Although the increase in plasma HOXD‐AS1 and RMRP levels in patients with NSCLC demonstrated statistically significant differences when compared with healthy controls (*p* = 0.038 and *p* = 0.032, respectively; Figure 2C,D), the degree of increase was inferior to that of CEA and CYFRA21‐1 (*p* = 0.012 and *p* = 0.02, respectively; Figure 2E,F). In addition, the increase in serum SCC‐A levels (*p* = 0.042) was less than that of CEA and CYFRA21‐1 (Figure 2E,F, and Figure S1). ROC analysis was conducted to evaluate the diagnostic performance of the four lncRNAs and three classic tumor markers for NSCLC detection. The AUC values for TP73‐AS1 and CRNDE were 0.822 and 0.815, respectively (Figure 2A,B), higher than those for CEA and CYFRA21‐1 (0.769 and 0.686, respectively; Figure 2E,F), whereas the AUC values of HOXD‐AS1 and RMRP (0.655 and 0.673, respectively) were lower than those of CEA and CYFRA21‐1 (Figure 2C,D). The AUC value of SCC‐A was the lowest among all biomarkers (0.653; Figure S1). Thus, HOXD‐AS1, RMRP, and SCC‐A were excluded from subsequent analyses. The detailed sensitivity, specificity, and accuracy of TP73‐AS1, CRNDE, CEA, and CYFRA21‐1, as well as their combined use for NSCLC diagnosis, are summarized in Table S5. On setting the specificity as 90%, plasma TP73‐AS1 showed the highest sensitivity and accuracy, followed by plasma CRNDE. These data indicated that plasma TP73‐AS1 and CRNDE are appropriate diagnostic biomarkers for NSCLC.

We then assessed the diagnostic efficiency of the two lncRNAs and two classic tumor markers in the training set. TP73‐AS1 plus CRNDE achieved a higher AUC value (0.884) compared with each lncRNA alone, with a sensitivity of 75% and an accuracy of 82% (Figure S2A and Table S5). The two lncRNAs plus CEA enhanced the AUC value to 0.922, with a sensitivity of 80% and an accuracy of 85% (Table S5), whereas the two lncRNAs plus CYFRA21‐1 did not further increase AUC and sensitivity values (data are not shown). The combination of the two lncRNAs and two tumor markers slightly increased the AUC value (0.927), sensitivity (82%), and accuracy (86%) when compared with the combination of the two lncRNAs and CEA (Figure S2A and Table S5).

### 3.4 Validation of the utility of plasma TP73‐AS1 and CRNDE for NSCLC diagnosis in the testing set

**FIGURE 3:**
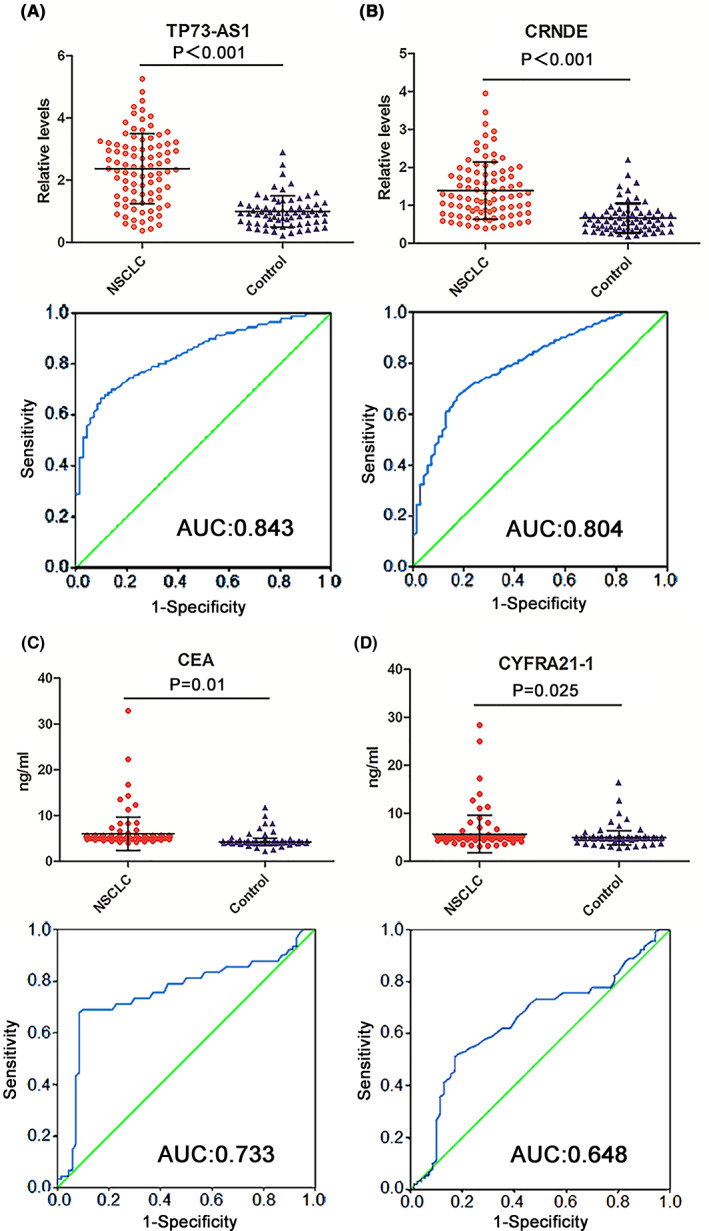
Expression levels and diagnostic performance of two plasma lncRNAs and two serum classic tumor markers in the testing set. (A) TP73‐AS1 and (B) CRNDE expressions are significantly elevated in the plasma of NSCLC patients compared with healthy controls. (C) CEA and (D) CYFRA21‐1 are markedly elevated in the serum of NSCLC patients compared with healthy controls (upper). The ROC curves and AUCs of TP73‐AS1 (A), CRNDE (B), CEA (C), and CYFRA21‐1 (D) in discerning NSCLC patients from healthy controls (lower). AUCs, areas under the ROC curves; CEA, carcinoembryonic antigen; CYFRA21‐1, cytokeratin 19 fragment; lncRNAs, long noncoding RNAs; NSCLC, non‐small cell lung cancer; ROC, receiver operating characteristic.

To further confirm the elevated expression of TP72‐AS1 and CRNDE and their diagnostic value for NSCLC, we measured their plasma levels by qRT‐PCR assay in another independent cohort of 90 patients with NSCLC and 70 healthy controls (testing set). Consistent with the training set, plasma levels of TP73‐AS1 and CRNDE were elevated in patients with NSCLC compared to those of healthy controls (both *p* < 0.001; Figure 3A,B). Serum levels of CEA and CYFRA21‐1 in the testing set are presented in Figure 3C,D. Likewise, AUC values of TP73‐AS1 and CRNDE (0.843 and 0.804) were higher than those of CEA and CYFRA21‐1 (0.733 and 0.648) in the testing set (Figure 3A–D). Moreover, the combination of TP73‐AS1 and CRNDE augmented the AUC value, sensitivity, and accuracy when compared with TP73‐AS1 and CRNDE alone for NSCLC diagnosis (Figure S2B and Table S6). Combining the two lncRNAs with CEA further increased diagnostic efficiency. The two lncRNAs, combined with the two tumor markers, displayed the best diagnostic efficiency (Figure S2B and Table S6).

### 3.5 Further evaluation of plasma TP73‐AS1 and CRNDE for NSCLC diagnosis in the extending set

**FIGURE 4:**
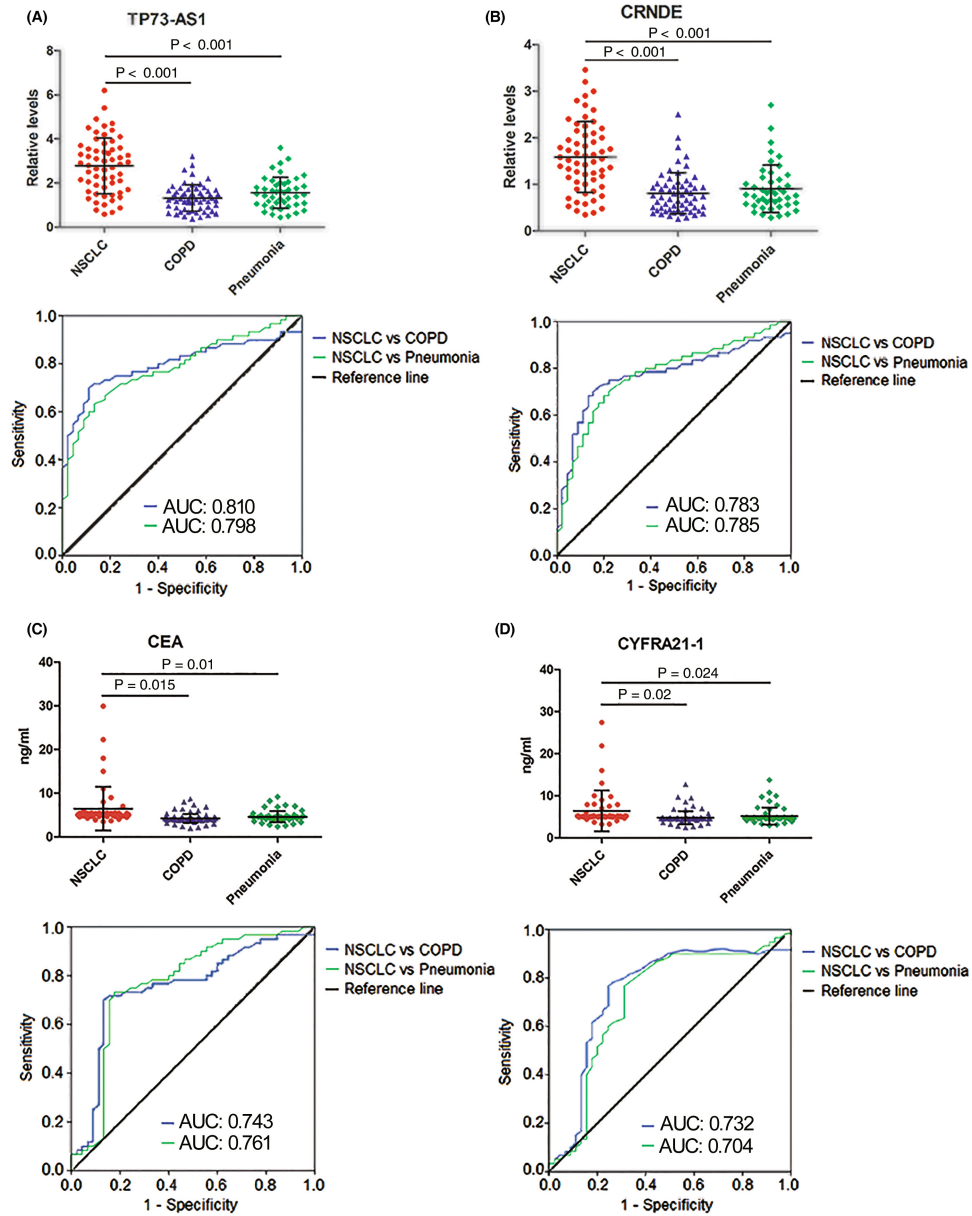
Expression levels and diagnostic performance of plasma TP73‐AS1 and CRNDE and serum CEA and CYFRA21‐1 in the extending set. Plasma levels of (A) TP73‐AS1 and **(**B) CRNDE and serum levels of (C) CEA and (D) CYFRA21‐1 are significantly elevated in NSCLC patients compared with patients with chronic obstructive pulmonary disease (COPD) and pneumonia (upper). The ROC curves and AUCs of TP73‐AS1 (A), CRNDE **(**B), CEA (C), and CYFRA21‐1 **(**D) in distinguishing NSCLC patients from patients with COPD and pneumonia (lower). AUCs, areas under the ROC curves; CEA, carcinoembryonic antigen; CYFRA21‐1, cytokeratin 19 fragment; lncRNAs, long noncoding RNAs; NSCLC, non‐small cell lung cancer; ROC, receiver operating characteristic.

To investigate the diagnostic efficiency of TP73‐AS1 and CRNDE in distinguishing NSCLC from BLD, an additional cohort of plasma specimens from 62 NSCLC patients, 60 patients with COPD, and 46 patients with pneumonia was utilized as an extension set. Similar to the results of training and testing sets, the extended set data showed significantly elevated plasma TP73‐AS1 and CRNDE levels in patients with NSCLC compared to patients with COPD and those with pneumonia (all *p* < 0.001; Figure 4A,B). ROC curve analyses revealed greater AUC values in discriminating NSCLC from COPD and pneumonia for TP73‐AS1 (0.810 for NSCLC vs. COPD and 0.798 for NSCLC vs. pneumonia) and CRNDE (0.783 for NSCLC vs. COPD and 0.785 for NSCLC vs. pneumonia) when compared with CEA (0.743 and 0.761, respectively) and CYFRA21‐1 (0.732 and 0.704, respectively; Figure 4C,D). These results demonstrated the greater ability of TP73‐AS1 and CRNDE to discern patients with NSCLC from those with BLD compared to CEA and CYFRA21‐1.

To estimate whether the expression levels of plasma TP73‐AS1 and CRNDE were related to clinicopathological features, we analyzed the correlation between clinicopathological parameters and the expression of the two plasma lncRNAs. In total, 212 patients with NSCLC in the three sets were stratified by high and low plasma lncRNA levels, based on median values. The results revealed that high TP73‐AS1 and CRNDE levels were positively correlated with tumor size, advanced TNM stage, and lymph node metastasis but were not associated with other characteristics in patients with NSCLC (Table S7 and Table S8).

### 3.6 Subgroup analyses of plasma TP73‐AS1 and CRNDE for NSCLC diagnosis

To explore whether TP73‐AS1 and CRNDE possess differential diagnostic capacities for different histological subtypes of NSCLC, we performed pairwise group comparisons of the two lncRNAs in three subgroups of all cases, consisting of training and testing sets, lung adenocarcinoma (LAD), lung squamous cell carcinoma (LSCC), and health controls. As presented in Figure S3A,B, although plasma levels of the two lncRNAs were higher in patients with LAD than in those with LSCC, the difference did not attain statistical significance. However, the two lncRNAs exhibited higher AUC values in discriminating patients with LAD from healthy controls relative to those differentiating between patients with LSCC and healthy controls (0.856 vs. 0.823 for TP73‐AS1 and 0.851 vs. 0.797 for CRNDE; Figure S2A,B).

We also evaluated differences in circulating levels of TP73‐AS1 and CRNDE based on the TNM stage of NSCLC by comparing circulating levels of the two lncRNAs in patients with stage I, stage II, and stage IIIA NSCLC and healthy control groups in the training and testing sets combined. We found that plasma TP73‐AS1 and CRNDE levels increased progressively in the three groups of patients with NSCLC, although some increased levels failed to demonstrate statistically significant differences (such as stage I vs. stage II and stage II vs. stage III A; Figure S2C,D). However, circulating levels of the two lncRNAs remained markedly higher in patients with stage I NSCLC than in healthy control groups (both *p* < 0.001; Figure S3C,D). We examined the diagnostic performance of the two lncRNAs in discriminating patients with NSCLC presenting different TNM stages from healthy controls. According to our results, although AUC values of TP73‐AS1 and CRNDE were relatively lower in patients with stage I (0.800 and 0.801) than stage II‐IIIA (0.839 and 0.837, and 0.871 and 0.861, respectively; Figure S3C,D), the AUCs of the two lncRNAs were still higher in stage I patients with NSCLC than those of CEA and CYFRA21‐1 in stage I‐IIIA patients with NSLC in training (0.769 and 0.686; Figure 2E,F) and testing (0.733 and 0.648; Figure 3C,D) sets. The findings signified that the two circulating lncRNA levels were elevated in stage I patients with NSCLC and exhibited excellent performance in discerning patients with early‐stage NSCLC from healthy controls.

### 3.7 Influence of plasma TP73‐AS1 and CRNDE expressions on TFS of patients with NSCLC after surgery

**FIGURE 5:**
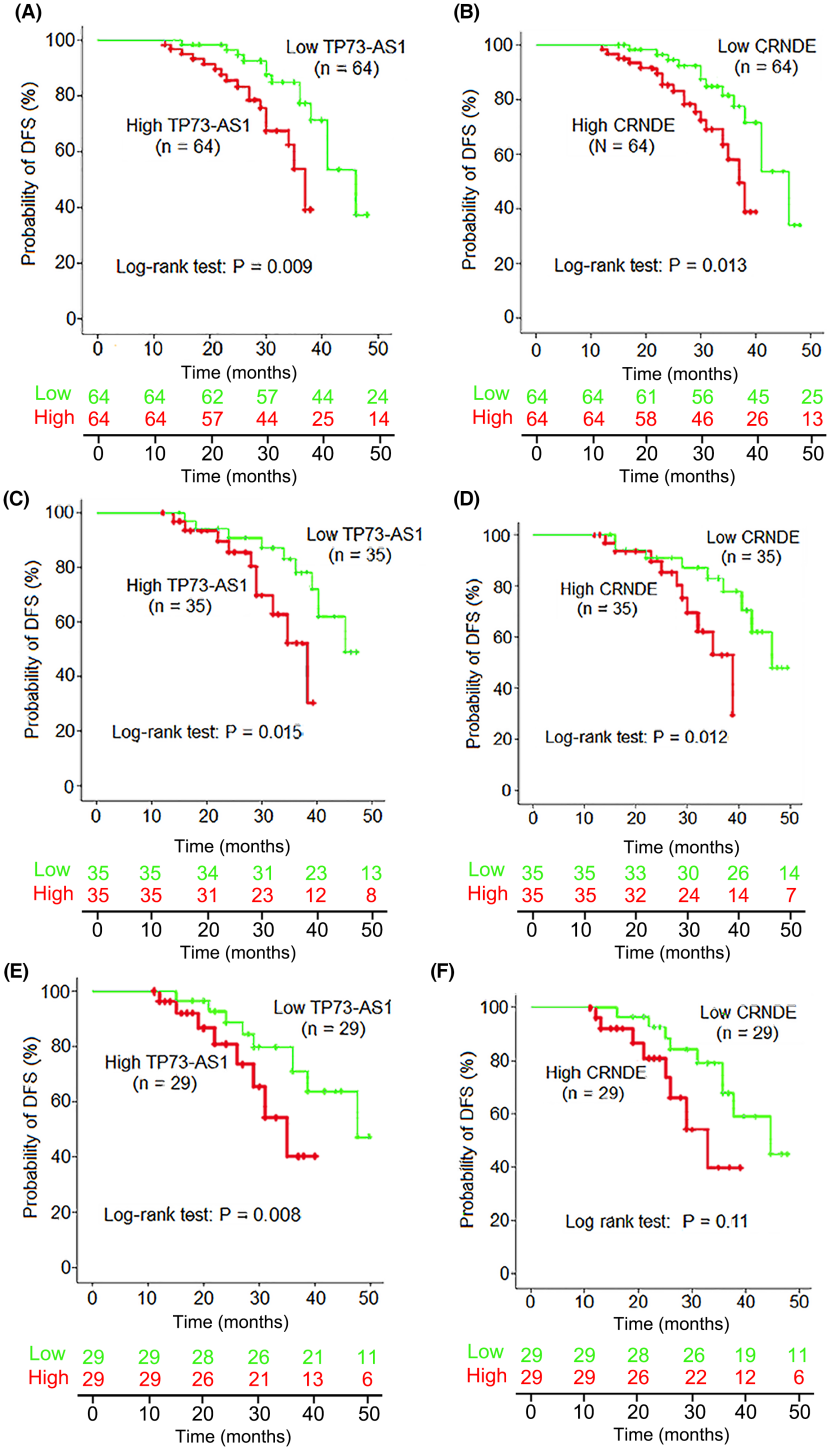
The correlation between the two plasma lncRNAs levels and tumor‐free survival (TFS) in NSCLC patients. (A, B) Kaplan–Meier curve analysis of TFS in NSCLC patients by high (green line) or low (red line) plasma levels of TP73‐AS1 and CRNDE. (C, D) Kaplan–Meier curve analyses of TFS in lung adenocarcinoma (LAD) patients and (E, F) lung squamous cell carcinoma (LSCC) patients by high (green line) or low (red line) plasma levels of TP73‐AS1 and CRNDE. lncRNAs, long noncoding RNAs; NSCLC, non‐small cell lung cancer.

We further analyzed the association between plasma levels of the two lncRNAs and TFS in patients with NSCLC. The Kaplan–Meier curve analysis was employed to examine the influence of plasma TP73‐AS1 and CRNDE expression on the TFS of 128 patients with NSCLC after surgery and adjuvant chemotherapy. The median follow‐up time was 42 months (range: 12–56 months). The median values for TP73‐AS1 and CRNDE levels in plasma samples were applied to divide patients with NSCLC into high‐ and low‐expression groups, and the difference in TFS between the two groups was analyzed using the log‐rank test. The data demonstrated that patients with high plasma TP73‐AS1 and CRNDE levels showed significantly poor TFS (*p* = 0.009 and *p* = 0.013, respectively; Figure 5A,B). Subgroup survival analysis revealed that elevated plasma TP73‐AS1 and CRNDE levels were markedly correlated with poor TFS in patients with LAD (*p* = 0.015 and *p* = 0.0012, respectively; Figure 5C,D) and LSCC (*p* = 0.008 and *p* = 0.011, respectively; Figure 5E,F).

Additionally, Cox proportional hazards model analysis was performed to further confirm the influence of plasma TP73‐AS1 and CRNDE expression on the TFS of patients with NSCLC. Univariate analyses revealed that poor TFS in patients with NSCLC was correlated with local lymph node metastasis (*p* = 0.006), TNM stage (*p* = 0.002), high plasma TP73‐AS1 (*p* < 0.001), and CRNDE levels (*p* < 0.001), as well as high serum CEA (*p* = 0.009) and CYFRA21‐1 levels (*p* = 0.015; Table S9). Parameters with a value of *p* < 0.05 in univariate analysis were then examined in a multivariate analysis. The results revealed that high plasma TP73‐AS1 and CRNDE levels, but not high serum CEA and CYFRA21‐1 levels, were independent predictive factors of poor TFS in patients with NSCLC (*p* = 0.006 for TP73‐AS1; *p* = 0.012 for CRNDE; Table S9).

### 3.8 Construction of a predictive nomogram and principal component analysis (PCA)

**FIGURE 6:**
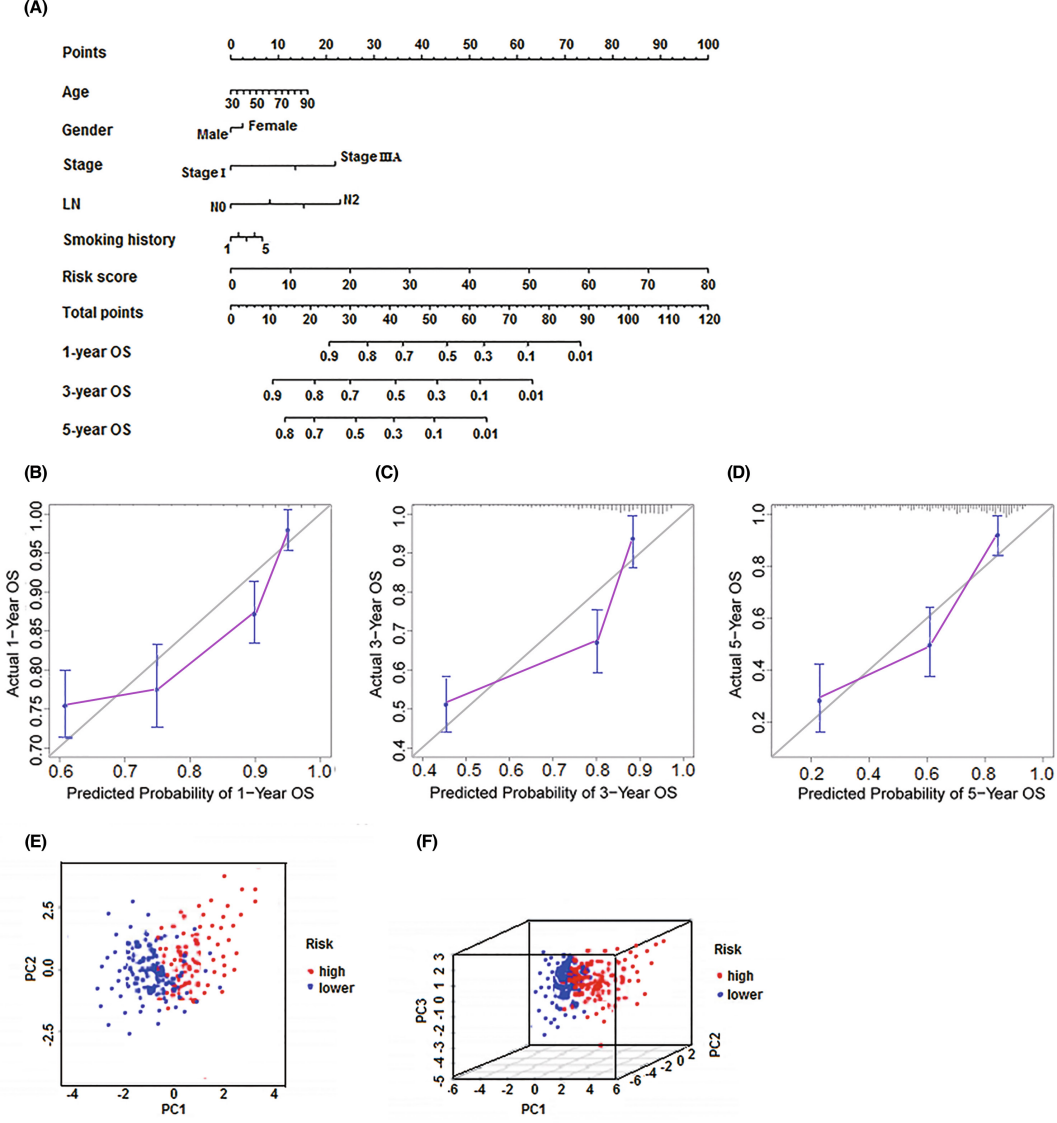
Clinical prognostic nomogram for overall survival prediction and principal component analysis. (A) A clinical prognostic nomogram was established to predict 1‐, 3‐, and 5‐year overall survival in NSCLC patients. (B–D) Calibration plots of the nomogram for calculating 1‐, 3‐, and 5‐year overall survival (E, F) Principal component analysis of the high‐ and low‐risk groups based on risk model including two prognosis‐related lncRNAs and two classic tumor markers. LN, lymph node; lncRNAs, long noncoding RNAs; NSCLC, non‐small cell lung cancer; OS, overall survival.

After selecting the prognosis‐related biomarkers through the above steps (Table S9), we performed a multivariate Cox proportional hazards regression analysis to calculate the coefficient of each biomarker. Significant prognosis‐related biomarkers (CEA and CYFRA21‐1) were integrated into a multivariate Cox regression analysis to generate risk scores. The risk score for each patient was calculated by the following formula: Risk score = ∑i coefficient (lncRNA1) × expression (lncRNA1) + coefficient (lncRNA2) × expression (lncRNA2) + coefficient (CEA) × expression (CEA) coefficient (CYFRA21‐1) × expression (CYFRA21‐1). We assessed the distribution of survival status according to the risk score levels. A clinically adaptable nomogram was constructed to evaluate the probability of 1‐, 3‐, and 5‐year overall survival (OS) in patients with NSCLC using the risk score and clinicopathological characteristics. The nomogram illustrated the contribution of each variable to the prediction of tumor‐related death at 1, 3, or 5 years (Figure 6A). The calibration plots illustrating the probability of 1‐, 3‐, and 5‐year OS after surgery displayed good uniformity between the predictions made by the nomogram and actual observations (B‐D). Then, a principal component analysis (PCA) was performed to compare the low‐ and high‐risk groups based on the risk model. As presented in Figure 6E,F, the low‐ and high‐risk groups had different distributions, suggesting that the prognostic signature can discriminate between low‐ and high‐risk groups.

### 3.9 TP73‐AS1 facilitates NSCLC cell proliferation and enhances cell migration and invasion abilities

**FIGURE 7:**
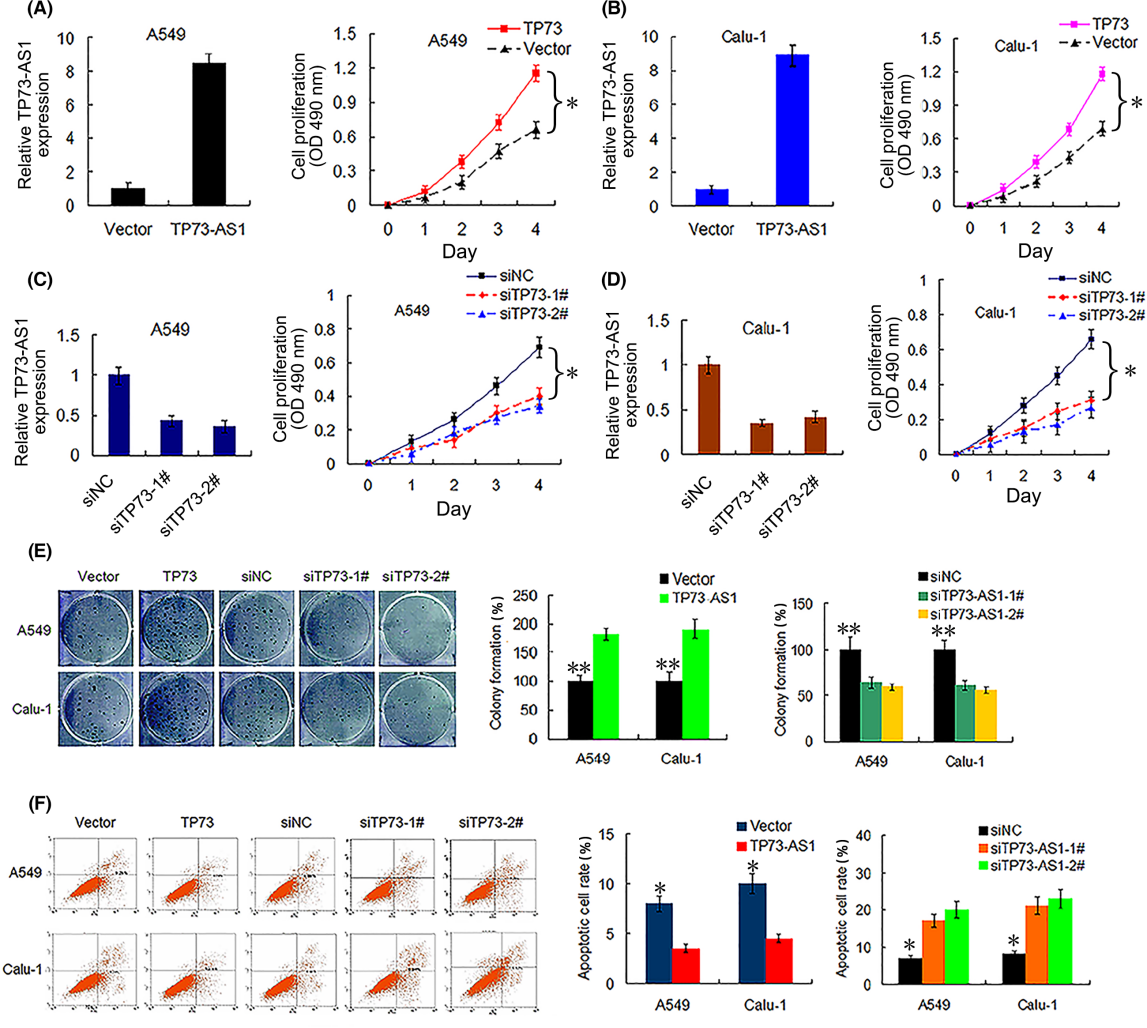
TP73‐AS1 promotes NSCLC cell viability and colony formation and suppresses cell apoptosis. (A, B) Transfection effectivity of TP73‐AS1 overexpression (left) and the influence of TP73‐AS1 expression on the cell viability (right, *p<0.01) of A549 and Cula‐1 cells, as determined by qRT‐PCR and CCK‐8 assays, respectively. (C, D) Transfection effectivity of TP73‐AS1‐siRNA (siTP73‐1# and siTP73‐2#, left), and the influence of TP73‐AS1 silencing on cell viability (right, *p<0.01) of A549 and Cula‐1 cells. (E) Overexpression (TP73) and knockdown (TP73‐1# and 2#) of TP73‐AS1 promotes and suppresses colony formation, respectively, in A549 and Cula‐1 cells (**p<0.001). (F) Overexpression (TP73) and silencing (siTP73‐1# and siTP73‐2#) of TP73‐AS1 represses and enhances cell apoptosis in A549 and Calu‐1 cells, respectively (*p<0.01). NSCLC, non‐small cell lung cancer; qRT‐PCR, quantitative reverse‐transcription PCR.

To gain insights into the biological function of TP73‐AS1 in NSCLC cells, we upregulated and knocked down TP73‐AS1 expression in A549 and Calu‐1 cells. Transfection efficiency was verified using qRT‐PCR assay (Figure 7A–D). First, we carried out cell viability and colony formation assays. As presented in Figure 7A,B, TP73‐AS1 overexpression potently enhanced the viability of A549 and Calu‐1 cells (both *p* < 0.01). Additionally, the upregulation of TP73‐AS1 dramatically augmented colony formation in the two cell lines (both *p* < 0.001; Figure 7E). Correspondingly, silencing TP73‐AS1 repressed cell growth (both *p* < 0.01; Figure 6C,D) and colony formation (both *p* < 0.001; Figure 7E) in the two examined cell lines. Flow cytometric analysis revealed that TP73‐AS1 overexpression suppressed the apoptosis of A549 and Calu‐1 cells, whereas TP73‐AS1 silencing increased the apoptosis of the two cell lines (all *p* < 0.01; Figure 7F).

**FIGURE 8:**
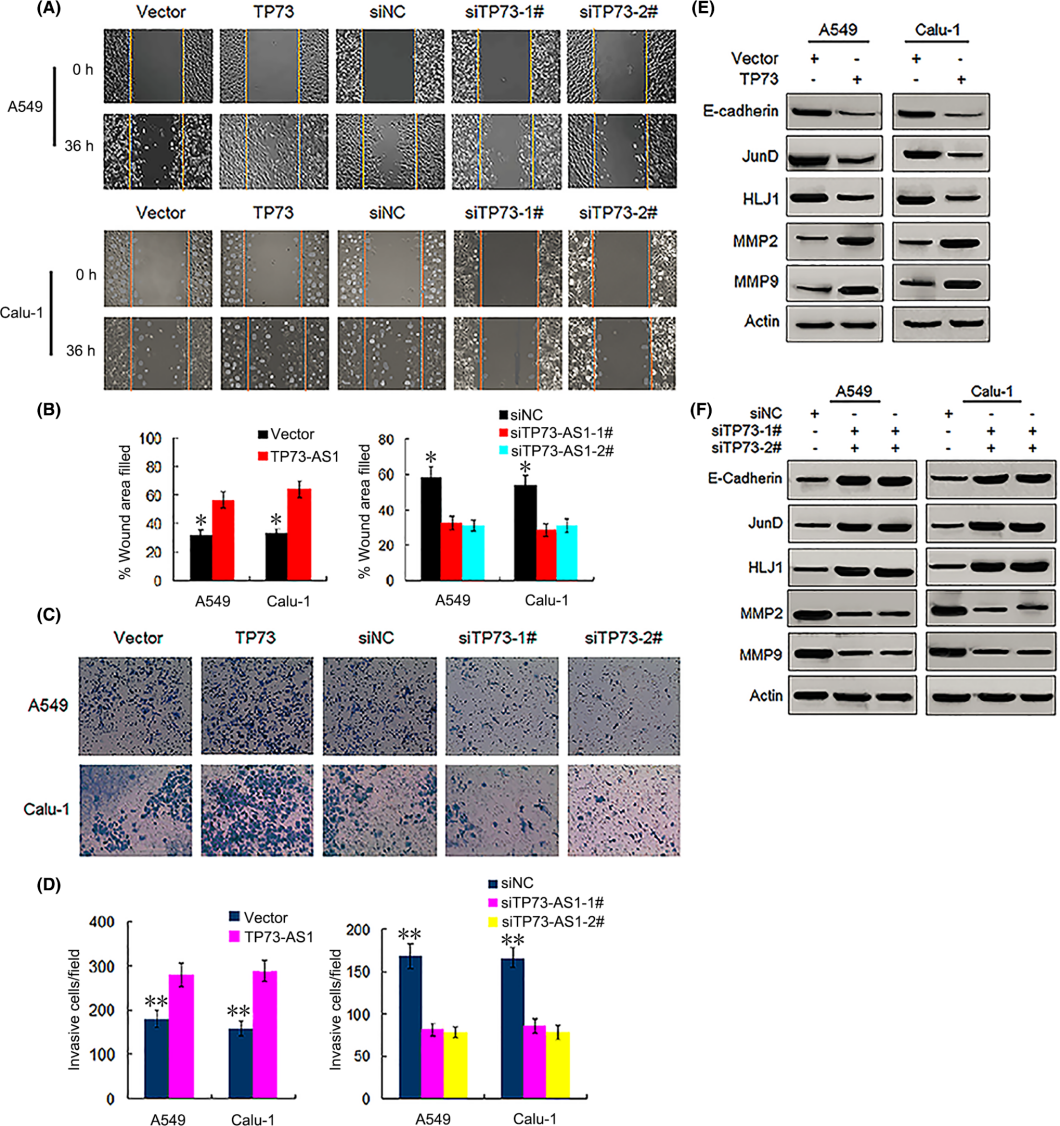
TP73‐AS1 augments migration and invasion capabilities of NSCLC cells. (A, B) Overexpression (TP73) and knockdown (siTP73‐1# and siTP73‐2#) of TP73‐AS1 promotes and suppresses migration abilities of A549 and Cula‐1 cells, respectively (* *p*<0.01). (C, D) Overexpression (TP73) and silencing (siTP73‐1# and siTP73‐2#) of TP73‐AS1 enhances and represses invasion abilities of A549 and Cula‐1 cells, respectively (**p<0.001). (E, F**)** Effects of overexpression (TP73) and silencing (siTP73‐1# and siTP73‐2#) of TP73‐AS1 on the protein expression of E‐cadherin, JunD, HLJ1, MMP2 and MMP9, as measured by western blotting. NSCLC, non‐small cell lung cancer.

We assessed the effect of TP73‐AS1 on the migration and invasion capabilities of A549 and Calu‐1 cells. According to the wound healing assay results, TP73‐AS1 overexpression significantly increased the percentage of wound area in A549 and Calu‐14 cells when compared with that in the vector group at 36 h, whereas knockdown of TP‐AS1 markedly decreased the percentage of wound area filled (all *p* < 0.01; Figure 8A,B), indicating that the upregulation of TP73‐AS1 facilitated cell migration. Transwell assays revealed that TP73‐AS1 overexpression enhanced cell invasion, whereas TP73‐AS1 silencing markedly decreased the invasion activity of the two cell lines (all *p* < 0.001; Figure 8C,D). These findings indicated that TP73‐AS1 facilitates cell proliferation and potentiates the migration and invasion abilities of NSCLC cells.

Additionally, we determined the expression of proteins involved in cell migration and invasion. Given that the upregulation of the tumor suppressor heat shock protein 4 (HLJ1) mediated by JunD, an activator protein (AP‐1) component,^18^, ^19^ can inhibit the migration and invasion of cancer cells via E‐cadherin,^20^ we examined the impact of TP73‐AS1 on the expression of these proteins. Western blotting showed that TP73‐AS1 overexpression notably suppressed the expression levels of HLJ1, JunD, and E‐cadherin and increased the expression of matrix metalloproteinase (MMP) 2 and MMP9, which play key roles in the degradation of the basement membrane collagen of cancer cells,^21^, ^22^ while TP73‐AS1 silencing resulted in the opposite effect (Figure 8E,F), thereby suggesting that TP73‐AS promoted cell migration and invasion, which might be mediated by repressing E‐cadherin, JunD, and HLJ1 and upregulating MMP2 and MMP9.

## 4 DISCUSSION

The prognosis and 5‐year survival rate of patients with NSCLC are intimately correlated with clinical stages, exhibiting a significant drop from 68–92% for patients with stage I/II NSCLC to 1%–13% for patients with stage III/IV NCSCL.^23^ The low 5‐year survival rate is primarily attributed to a lack of effective tools for early detection of NSCLC, and therefore, there is an unmet demand to develop a rational approach for early NSCLC detection, which would greatly facilitate early intervention. Recently, accumulating evidence has shown that lncRNAs can be measured in the blood of patients with cancer and thus may be employed for cancer detection.^6^, ^7^ Some investigators have focused on circulating lncRNAs as biomarkers for NSCLC diagnosis and prognostic prediction.^13^, ^14^, ^24^ However, few systematic investigations have examined the possible application of circulating lncRNA quantitation for discriminating patients with NSCLC from healthy controls or patients with BLD, as well as for predicting TFS of patients with NSCLC after surgery.

In this study, we first selected 14 candidate lncRNAs that were found to be aberrantly expressed in NSCLC tissues and cells and play an key role in carcinogenesis in recent reports (Table S1). Subsequently, we investigated their potential application as circulating biomarkers for NSCLC detection. We found that TP73‐AS1 and CRNDE levels were notably increased in the plasma of patients with NSCLC, containing patients with early‐stage NSCLC, compared with healthy controls in training and testing sets. We also observed that plasma TP73‐AS1 and CRNDE levels were markedly elevated in patients with NSCLC compared with patients with BLD, including patients with COPD and pneumonia in an extended set. Moreover, we demonstrated that plasma TP73‐AS1 and CRNDE have better diagnostic efficiency for NSCLC than the classic tumor markers CEA and CYFRA21‐1. Importantly, we observed that the two plasma lncRNAs showed remarkable diagnostic efficiency for patients with stage I and stage II/IIIA NSCLC; thus, they could be employed for detecting both early‐ and late‐stage NSCLC. In addition, plasma TP73‐AS1 and CRNDE were equally effective for detecting both LAD and LSCC, suggesting that the diagnostic efficiency of the two lncRNAs is independent of the histological subtype of NSCLC.

In several types of cancer, the combination of multiple markers has been shown to improve their diagnostic performance for cancer detection.^25^, ^26^ Therefore, we combined the two lncRNAs and classic tumor markers CEA and CYFRA21‐1 through a logistic regression model to assess their diagnostic performance for NSCLC. As expected, the combination of TP73‐AS1 and CRNDE displayed greater diagnostic efficiency than either of the two lncRNAs alone. Combining the two lncRNAs with CEA further improved diagnostic performance. Although the two lncRNAs in combination with CYFRA21‐1 did not afford better results than the combination of the two lncRNAs alone, the four marker combinations further improved diagnostic performance in the training and testing sets. These data indicate that TP73‐AS1 and CRNDE might be valuable biomarkers for diagnosing NSCLC and that the two lncRNAs in combination with CEA or CEA/CYFRA21‐1 should be an ideal diagnostic panel for NSCLC.

Herein, it should be noted that plasma TP73‐AS1 and CRNDE levels markedly correlated with TFS in patients with NSCLC after surgery. Kaplan–Meier curve analyses showed that patients with NSCLC exhibiting high plasma levels of TP73‐AS1 or CRNDE had markedly shorter DFS than those with low plasma levels. Furthermore, univariate and multivariate analyses identified plasma TP73‐AS1 and CRNDE as independent predictive factors for TFS in NSCLC, suggesting an important role for the two lncRNAs in prognostic prediction. Considering that surgery is the first‐line recommended therapy in patients with stage I‐IIIA NSCLC, the signature of the two lncRNAs could help physicians assess patient prognosis after surgery and implement effective treatment options. In addition, studies have described similar findings indicating the effect of TP73‐AS1 and CRNDE expression in NSCLC tissues on the prognosis of patients with NSCLC. Zhang et al reported that patients with NSCLC exhibiting TP73‐AS1 overexpression in the tumor tissue had notably poor OS.^8^ Jing et al observed that high expression of CRNDE in NSCLC tissues can be markedly associated with worse OS of patients.^27^ These studies indicate that overexpression of TP73‐AS1 and CRNDE in cancer tissues can be correlated with advanced TNM stage and local lymph node metastasis in patients with NSCLC.^8^, ^27^ These results imply that TP73‐AS1 and CRNDE might serve as proto‐oncogene involved in the carcinogenesis of NSCLC. However, these studies did not examine the potential of the two circulating lncRNAs as biomarkers for the diagnosis and prediction of TFS.^8^, ^27^

Furthermore, we constructed a nomogram using the risk status combined with other clinicopathological characteristics to identify high‐risk patients with NSCLC, and calibration plots revealed that the actual observed versus nomogram‐predicted rates of 1‐, 3‐, and 5‐year OS showed good consistency. In addition, we applied the PCA method to examine different distribution patterns between low‐ and high‐risk patient groups according to the risk model. The low‐ and high‐risk groups were divided into two parts based on risk scores. These findings suggest that risk evaluation scores can contribute to the identification of high‐risk patients from patients exhibiting identical clinicopathological characteristics. Recent studies have also indicated that bioinformatic analysis can effectively identify prognosis‐related lncRNAs in NSCLC and other types of cancers using nomogram and PCA, thereby constructing the prognostic signature that provides a new strategy for predicting prognosis in patients with cancer.^28^, ^29^, ^30^, ^31^, ^32^

Elucidating the underlying molecular functions of lncRNAs in carcinogenesis would promote their application in clinical settings. Using in vitro experiments, we revealed that upregulated TP73‐AS1 expression markedly facilitated NSCLC cell proliferation and inhibited apoptosis. Furthermore, TP73‐AS1 augmented the migration and invasion abilities of NSCLC cells by depressing the expression levels of E‐cadherin, JanD, and HLJ1 while enhancing MMP2 and MMP9 expression. Silencing of TP73‐AS1 led to contradictory results. HLJ1 was found to be a tumor suppressor and a selected candidate target for suppressing metastasis and invasion.^18^, ^33^ E‐cadherin, as a crucial component for regulation and control of epithelial‐mesenchymal transition, reportedly functions as a cancer metastasis suppressor, and cancer cells with downregulated E‐cadherin expression are more likely to separate from a tumor mass, resulting in metastasis.^20^, ^34^ HLJ1 can reportedly modulate tumor cell migration and invasion via E‐cadherin.^20^ MMP2 and MMP9 have been demonstrated to play critical roles in the degradation of basement membrane collagen, as well as cancer progression and metastasis.^21^, ^22^ In addition, studies have reported results consistent with our findings.^8^, ^35^, ^36^ Zhang et al have reported that TP73‐AS1 silencing can repress in vitro NSCLC cell viability and cycle progression and suppress in vivo tumor growth by competitively sponging miR‐449a/EZHZ.^8^ The TP73‐AS1/miR‐449a/EZHZ pathway can promote NSCLC carcinogenesis via epigenetic modulation.^8^ The loss/gain‐of‐function assays performed by Liu et al have shown that TP73‐AS1 contributed to LAD cell survival, invasion, and migration in vitro, and its knockdown restrained LAD tumor growth and metastasis in vivo via upregulation of the PI3K/AKT pathway.^35^ In ovarian cancer, TP73‐AS1 was showed to promote cell survival and metastasis via increasing MMP2 and MMP9 expressions.^34^ Likewise, some researchers have reported the effect of CRNDE on carcinogenesis of NSCLC cells. For example, the silencing of CRNDE markedly suppressed NSCLC cell proliferation and clonogenic invasion and migration abilities in vitro and inhibited tumor xenograft growth in vivo by sponging miR‐338‐3P.^27^ High CRNDE expression increased NSCLC cell proliferation in vitro, promoted NSCLC tumor growth in vivo, and accelerated the cell cycle transition from G0/G1 stage to M stage via activation of the PI3K/AKT pathway.^37^

In conclusion, the data of this study indicate that plasma TP73‐AS1 and CRNDE levels could be applied for discriminating NSCLC from healthy controls or BLD and could be potentially used to diagnose early‐stage NSCLC. In addition, our study provides robust evidence that the two circulating lncRNAs can be novel predictive markers of TFS in patients undergoing surgery. Further validation investigations using a large sample of patients are needed to confirm the diagnostic and prognostic value and clinical utility of the two lncRNAs in patients with NSCLC.

## AUTHOR CONTRIBUTIONS

R‐XY, C‐HD, and JL conceived and designed the study. R‐XY, PC, and M‐JL collected the specimens and data. R‐XY, PC, M‐JL, YS, Z‐PW, and Y‐PX performed experiments. R‐XY, C‐HD, and Z‐PW acquired and analyzed the data. YS provided technical and material support. C‐HD and JL drafted and revised the manuscript. All authors have reviewed and approved the final manuscript.

## FUNDING INFORMATION

This study was supported by the Medical Research Program of Jiangsu Health Committee in China (Grant No. ZDB2020022) and The Social Development Foundation of Zhenjiang in China (Grant No. SH2014076, SH2015063).

## CONFLICT OF INTEREST

The authors have no conflict of interest to declare.

## CONSENT FOR PUBLICATION

Not applicable.

## ETHICS APPROVAL AND CONSENT TO PARTICIPATE

This study was approved by the Ethics Review Board of the Affiliated Hospital of Jiangsu University (No. JDFY‐2015029), China and in accordance with the ethical standards of the institutional and the Declaration of Helsinki. Informed consent was obtained from all participants.

## Supporting information


Figure S1‐S3

Table S1‐S9

## Data Availability

The data that support the findings of this study are available from the corresponding author on reasonable request.
