# Supplementary material for: Circulating TP73‐AS1 and CRNDE serve as diagnostic and prognostic biomarkers for non‐small cell lung cancer
Source: Cancer Med. 2022 Jul 24;12(2):1655–72. doi: 10.1002/cam4.5013 (PMC9883423; doi:10.1002/cam4.5013)
Supplement: Supplementary file 1 — Figure S1‐S3 [file CAM4-12-1655-s002.pdf]

Supplementary Figures

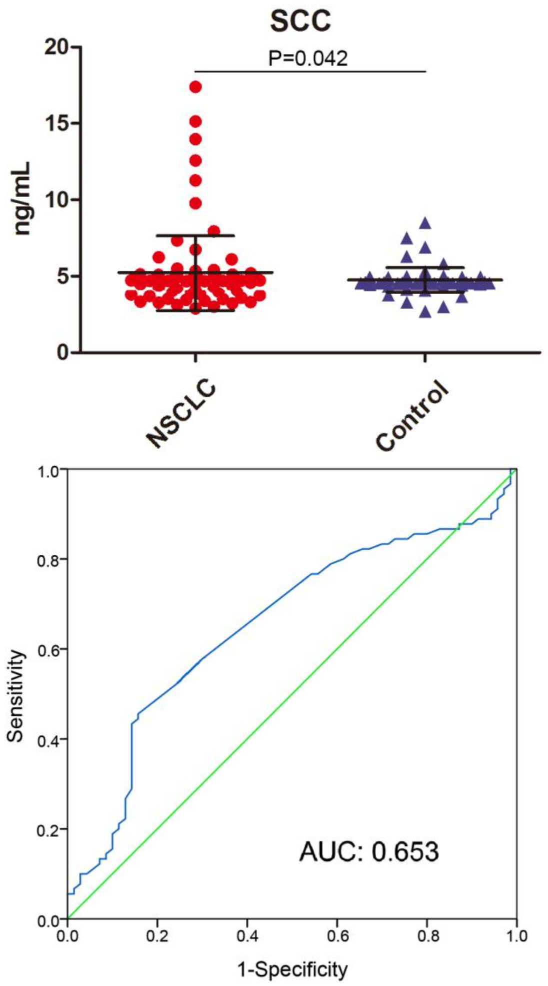

Figure S1 Expression levels and diagnostic efficiency of serum SCC-A. Serum SCC-A level were markedly elevated in patients with NSCLC compared with healthy controls (upper). The ROC curve and the AUCs of SCC-A in distinguishing NSCLC from healthy control (lower)

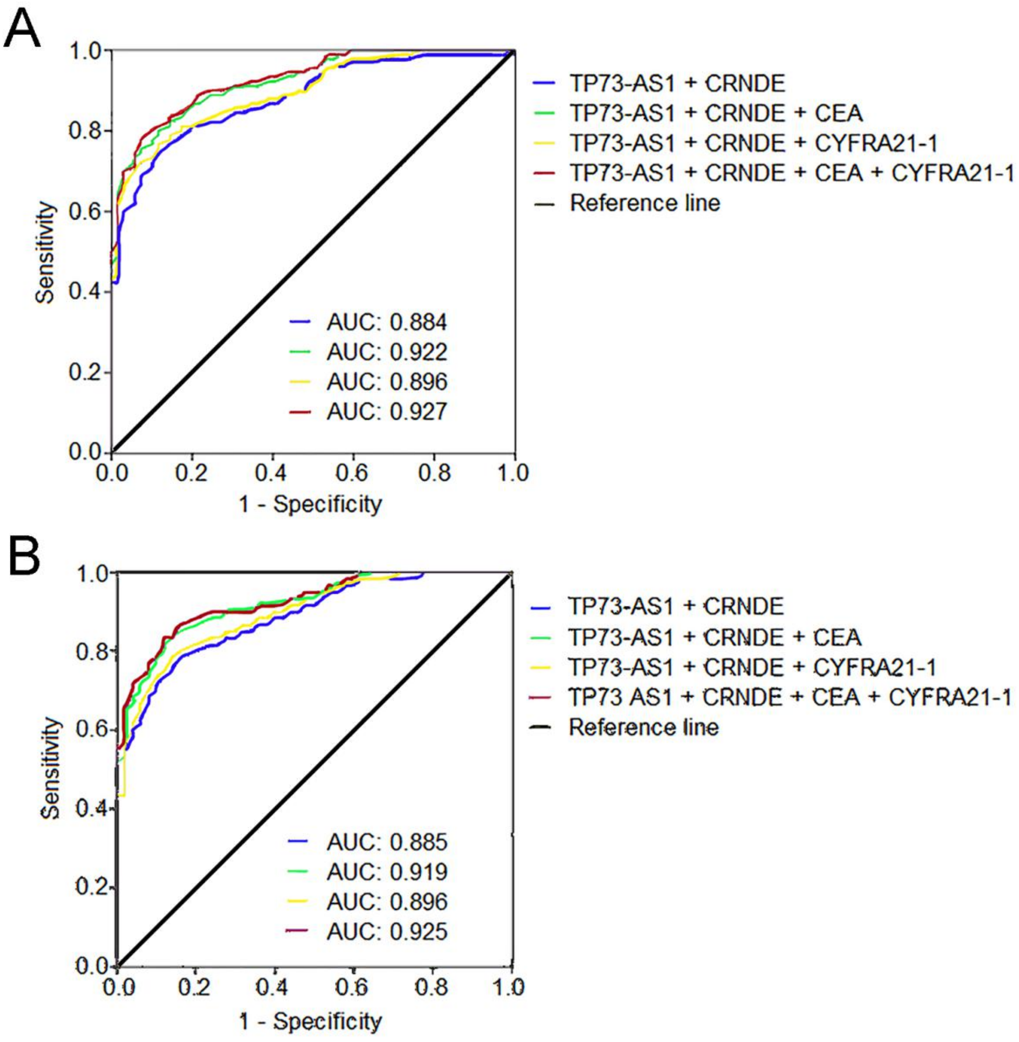

Figure S2 Analyses of receiving-operating characteristic (ROC) curves of the combination of plasma TP73-AS1 and CRNDE, and the combination of the two plasma lncRNAs and serum CEA or CYFRA21-1 in training set (**A**) and testing set (**B**), and the AUC values of these combinations in distinguishing NSCLC from healthy control.

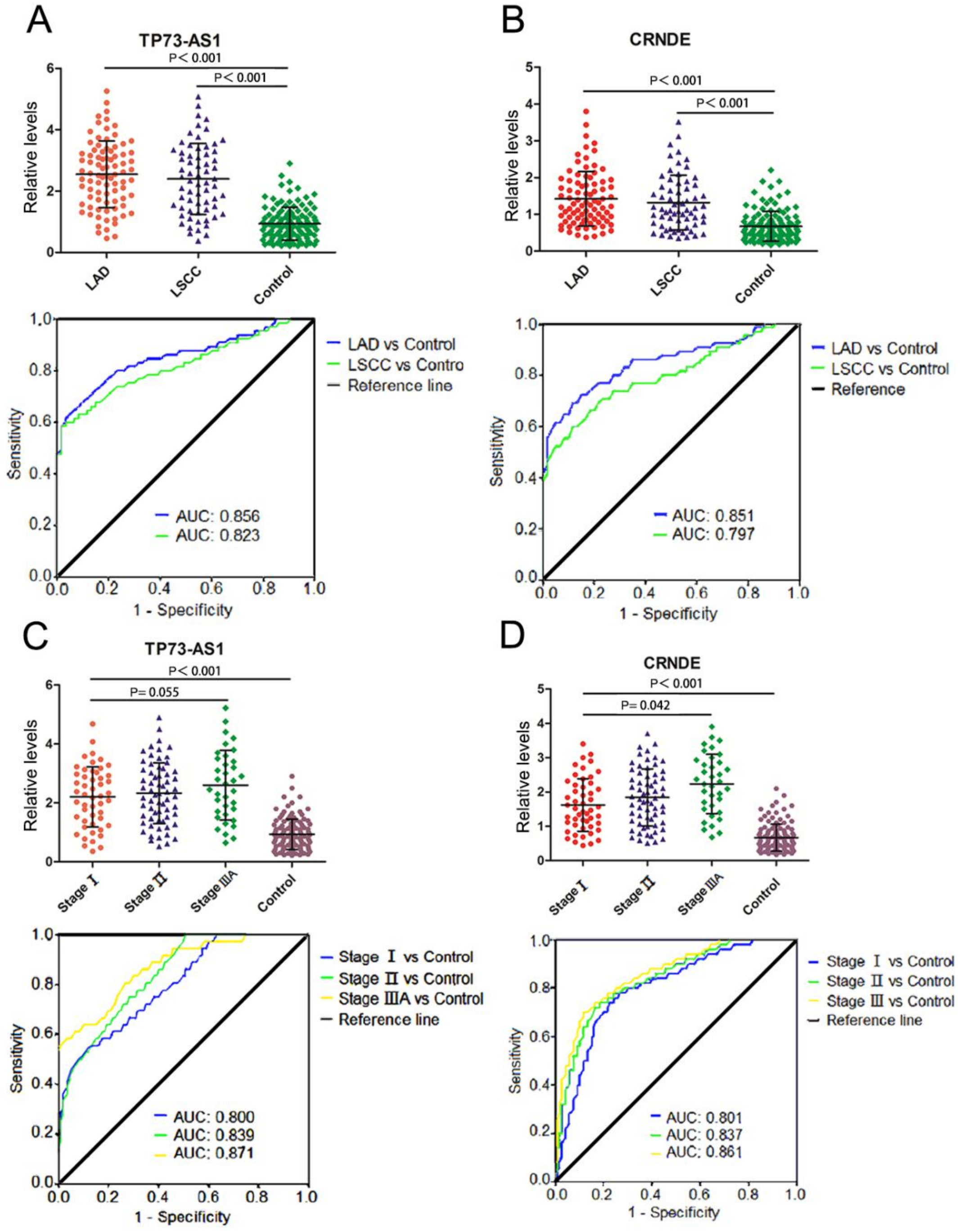

Figure S3 Plasma levels and diagnostic performance of TP73-AS1 and CRNDE in NSCLC patients with different histological subtypes and different TNM stages. (**A** and **B**) TP73-AS1 and CRNDE were significantly upregulated in the plasma of patients with lung adenocarcinoma (LAD) and patients with lung squamous cell carcinoma (LSCC) compared to healthy controls (upper). The AUC of TP73-AS1 and CRNDE in LAD patients were slightly higher than those in LSCC patients (lower). (**C**

and **D**) TP73-AS1 and CRNDE were significantly increased in the plasma of patients with stage Ⅰ, stage Ⅱ and stage ⅢA NSCLC as compared with healthy controls (upper). The AUC of TP73-AS1 and CRNDE in patients with stage Ⅰ NSCLC patients were slightly lower than those in patients with stage Ⅱ-ⅢA NSCLC (lower).
