# Supplementary material for: Circulating TP73‐AS1 and CRNDE serve as diagnostic and prognostic biomarkers for non‐small cell lung cancer
Source: Cancer Med. 2022 Jul 24;12(2):1655–72. doi: 10.1002/cam4.5013 (PMC9883423; doi:10.1002/cam4.5013)
Supplement: Supplementary file 2 — Table S1‐S9 [file CAM4-12-1655-s001.docx]

**Supplementary Table S1**

**Reported lncRNAs aberrantly expressed in NSCLC tissues (References)**

| **lncRNA** | **Deregulated in NSCLC** | **References** |
| --- | --- | --- |
| TP73-AS1 | Up | [1, 2] |
| CRNDE | Up | [ 3 ] |
| HOXP-AS1 | Up | [4, 5] |
| RMRP | Up | [6, 7] |
| TMPO-AS1 | Up | [8, 9] |
| HEIH | Up | [ 10 ] |
| SNHG1 | Up | [11, 12] |
| PART1 | Up | [13, 14] |
| NEAT1 | Up | [15, 16] |
| TUG1 | Down | [15, 17] |
| GAS5 | Down | [15, 18] |
| FAS-AS1 | Down | [ 15 ] |
| HOTAIRM1 | Down | [15, 19] |
| THRIL | Down | [ 15 ] |

**References**

1. Zhang L, Fang F, He X. Long noncoding RNA TP73-AS1 Promotes non-small cell lung cancer progression by competitively sponging miR-449 a/EZH2. Biomed Pharmacother. 2018;104:705-11.
2. Zhu D, Zhou J, Liu Y, et al. lncRNA TP73-AS1 is upregulated in non-small cell lung cancer and predicts poor survival. Gen 2019;710:98-102.
3. Liu X-X, Xiong H-P, Huang J-S, et al. Highly expressed long non-coding RNA CRNDE promotes cell proliferation through PI3K/AKT signalling in non-small cell lung cancer. Clin Exp Pharmacol Physiol 2017;44:489-902.
4. Wang Q, Jiang S, Song A, et al. HOXD-AS1 functions as an oncogenic ceRNA to promote NSCLC cell progression by sequestering miR-147a. Oncotarget Ther. 2017;10:4753-63.
5. Xia H, Ting H, Li Y, et al. Long noncoding RNA HOXD-AS1 promotes non-small cell lung cancer migration and invasive thorough regulating miR-1336/MMP9 axis. Biomed Pharmacother. 2018;106:156-62.
6. Lin Y, Leng Q, Zhan M, et al. A plasma long non-coding RNAs signature forn early detection of lung cancer. Tansl Oncol 2018; 11:1225-1231.
7. Meng Q, Ren M, Li Y, et al. lncRNA-RMRP acts as an oncogene in lung cancer. PLOs ONe. 2016;11:e0164845.
8. Li D-S, Ainiwar J-L, Sheyhiding I, et al. Identification of key long non-coding RNAs as competing endogenous RNAs for miRNA-mRNA in lung adenocarcinoma. Eur Rev Med Pharmacol Sci 2016;20: 2285-2295.
9. Peng F, Wang R, Zhang Y, et al. Differential expression analysis at the individual level reveals a lncRNA prognostic signature for lung adenocarcinoma. Mol Cancer 2017;16: 98.
10. Jia K, Chen F, Xu L. Long noncoding RNA HEIH promotes the proliferation and metastasis of non-small cell lung cancer. J Cell Biochem. 2019;120:3529-38.
11. Zhang H-Y, Yang W, Zheng F-S, et al. Long non-coding RNA SNHG1 regulates zinc finger E-boxbinding homeobox/expression by interacting with TAp63 and promotes cell metastasis and invasion in lung squamous cell carcinoma. Biomed Pharmacother. 2017;90:650-8.
12. Lu Q, Shan S, Li Y, et al. Long noncoding RNA SNHG1 promotes non-small cell lung cancer progression by up-regulating MTDH via sponging miR-145-5p. FASEB J. 2018;32:3957-67.
13. Li M, Zhang W, Zhang S, et al. PART1 expression is associated with poor prognosis and tumor recurrence in stageⅠ-Ⅲ non-small cell lung cancer. J Cancer. 2017;8:1795-800.
14. Zhu D, Yu Y, Wang W, et al. Long noncoding RNA PART1 promotes progression of non-small cell lung cancer cells via JAK-STAT signaling pathway. Cancer Med. 2019;8:6064-81.
15. Esfandi F, Taheri M, Omrani MD, et al. Expression of long non-coding RNA (lncRNAs) has been dysregulated in non-small cell lung cancer tissues. BMC Cancer. 2019;19:222.
16. Sun C, Li S, Zhang F, et al. Long non-coding RNA NEAT1 promotes non-small cell lung cancer progression through regulation of miR-377-3p-E2F3 pathway. Oncotarget 2016;7:51784-51814.
17. Lin P-C, Huang H-D, Chang C-C, et al. Long noncoding RNA TUG1 is downregulated in non-small cell lung cancer and can regulate CELF1 on binding to PRC2. BMC Cancer. 2016;16:583.
18. Mei Y, Si J, Wang Y, et al. Long noncoding RNAGAS5 suppresses tumorigenesis by inhibiting miR-23a expression in non-small cell lung cancer. Oncol Res 2017;25:1027-37.
19. Xiong F, Yin H, Zhang H, et al. Clinicopathologic feature and the prognostic implication of long noncoding RNA HOTAIRM1 in non-small cell lung cancer. Genet Test Mol Biomarkers. 2020;24:47-53.

**Supplementary Table S2**

**The 14 lncRNA expression levels in NSCLC tissues and adjacent normal lung tissues**

| **lncRNA** | **NSCLC** | **Normal** | **Change** | ***P* value** |
| --- | --- | --- | --- | --- |
|  | **tissue** | **lung tissue** | **fold** |  |
| TP73-AS1 | 1.97 (1.12-3.02) | 0.92 (0.45-1.76) | 2.14 | ＜ 0.001 |
| CRNDE | 1.45 (0.95-1.89) | 0.67 (0.25-1.04) | 2.15 | ＜ 0.001 |
| HOXD-AS1 | 1.55 (0.73-2.38) | 0.76 (0.34-1.19) | 2.04 | ＜ 0.001 |
| RMRP | 1.72 (0.94-2.45) | 0.85 (0.42-1.27) | 2.02 | ＜ 0.001 |
| TMPO-AS1 | 1.97 (0.92-2.64) | 1.26 (0.74-1.59) | 1.56 | 0.004 |
| HEIH | 1.80 (0.88-2.66) | 1.25 (0.82-1.67) | 1.44 | 0.005 |
| SNHG1 | 1.76 (1.18-2.28) | 1.32 (0.77-1.96) | 1.33 | 0.012 |
| PART1 | 1.98 (1.24-2.83) | 1.40 (0.91-1.94) | 1.41 | 0.008 |
| NEAT1 | 1.68 (1.07-2.12) | 1.23 (0.77-1.82) | 1.37 | 0.015 |
| TUG1 | 1.71 (1.15-2.42) | 1.68 (0.84-2.05) | 1.20 | 0.119 |
| GAS5 | 1.82(1.47-2.21) | 1.49 (0.73-1.97) | 1.22 | 0.115 |
| FAS-AS1 | 1.94(1.51-2.36) | 1.47 (0.81-1.99) | 1.32 | 0.014 |
| HOTAIRM1 | 2.21(1.82-2.64) | 1.74 (1.02-2.34) | 1.27 | 0.124 |
| THRIL | 2.34(1.98-2.89) | 2.01 (1.56-2.65) | 1.16 | 0.132 |

**Supplementary Table S3**

**The primer sequences of candidate lncRNAs chosen from previous NSCLC studies**

| **Gene name** | **Primer sequence** |
| --- | --- |
| TP73-AS1 | F:5’-TCATTTCTGCCCCTACTCCT-3’ |
|  | R:5’-CAGTCACATGTCTGCGCTAAT-3’ |
| CRNDE | F:5’-ATATTCAGCCGTTGGTCTTTGA-3’ |
|  | R:5’-TCTGCGTGACAACTGAGGATTT-3’ |
| HOXD-AS1 | F:5’-CCTTGAAAGTGGGTAAAATGTGC-3’ |
|  | R:5’-TAGTTTCCTTGTTCCTTTGTGCTGT-3’ |
| RMRP | F:5’-ACTCCAAAGTCCGCCAAGA-3’ |
|  | R:5’-TGCGTAACTAGAGGGAGCTGAC-3’ |
| TMPO-AS1 | F:5’-AGCCAGACCTCTACAATCGG-3’ |
|  | R:5’-TTAGGATTCTTGCGGGTGGT-3’ |
| HEIH | F:5’-CCTCTTGTGCCCCTTTCT-3’ |
|  | R:5’-AGGTCTCATGGCTTCTCG-3’ |
| SNHG1 | F:5’-TAACCTGCTTGGCTCAAAGGG-3’ |
|  | R:5’-CAGCCTGGAGTGAACACAGA-3’ |
| PART1 | F:5’-AAGGCCGTGTCAGAACTCAA-3’ |
|  | R:5’-GTTTTCCATCTCAGCCTGGA-3’ |
| NEAT1 | F:5’-CCAGTGTGAGTCCTAGCATTGC-3’ |
|  | R:5’-CCTGGAAACAGAACATTGGAGAAC-3’ |
| TUG1 | F:5’-ACCGGAGGAGCCATCTTGTC-3’ |
|  | R:5’-GAAAGAGCCGCCAACCGATC-3’ |
| GAS5 | F:5’-CTGCTTGAAAGGGTCTTGCC-3’ |
|  | R:5’-GGAGGCTGAGGATCACTTGAG-3’ |
| FAS-AS1 | F:5’-GAAAAGGTGCCCTTTCTTCCG-3’ |
|  | R:5’-CTGGCAGTTCTCAGACGTAGG-3’ |
| HOTAIRM1 | F:5’-GAAGAGCAAAAGCTGCGTTCTG-3’ |
|  | R:5’-CTCTCGCCAGTTCATCTTTCATTG-3’ |
| THRIL | F:5’-GAGTGCAGTGGCGTTGATCTC-3’ |
|  | R:5’-AAAATTAGTCAGGCATGGTGGTG-3’ |

**Supplementary Table S4**

**Characteristics of subjects in extending set**

|  | **NSCLC** | **COPD** | **Pneumonia** | ***P* value** |
| --- | --- | --- | --- | --- |
|  | **(n=62)** | **(n=60)** | **(n=46)** |  |
| Age (years) |  |  |  |  |
| Mean ± SD | 62.2±9.7 | 67.4±7.5 | 52.3±14.5 | 0.017 |
| Gender |  |  |  |  |
| Male | 34(55%) | 41(68%) | 27(59%) | 0.176 |
| Female | 28(45%) | 19(32%) | 19(41%) |  |
| Smoking status |  |  |  |  |
| Nonsmoker | 27(43%) | 20(33%) | 28(61%) | 0.007 |
| Smoker | 35(57%) | 40(67%) | 18(39%) |  |
| Histology |  |  |  |  |
| Adenocarcinoma | 36(58%) | NA | NA |  |
| SCC | 26(42%) | NA | NA |  |
| TNM stage |  |  |  |  |
| Ⅰ | 25(40%) | NA | NA |  |
| Ⅱ | 27(44%) | NA | NA |  |
| ⅢA | 10(16%) | NA | NA |  |

NSCLC, non-small cell lung cancer; COPD, chronic obstructive pulmonary disease; SCC, squamous cell carcinoma; TNM, tumor node metastasis; NA, not assessed.

**Supplementary Table S5**

**Efficiency of four markers and their combined use in diagnosis of NSCLC in training set**

| **Marker** | **AUC** | **95% CI** | **Sensitivity** | **Specificity** | **Accuracy** |
| --- | --- | --- | --- | --- | --- |
| TP73-AS1 | 0.822 | 0.64-0.95 | 67% | 90% | 77% |
| CRNDE | 0.815 | 0.60-0.93 | 63% | 90% | 75% |
| CEA | 0.769 | 0.53-0.89 | 60% | 90% | 74% |
| CYFRA21-1 | 0.686 | 0.48-0.88 | 52% | 90% | 69% |
| TP73-AS1+CRNDE | 0.884 | 0.70-1.05 | 75% | 90% | 82% |
| TP73-AS1+CRNDE+CEA | 0.922 | 0.79-1.22 | 80% | 90% | 85% |
| TP73-AS1+CRNDE+CEA+CYFRA21-1 | 0.927 | 0.79-1.22 | 82% | 90% | 86% |

**Supplementary Table S6**

**Efficiency of four markers and their combined use in diagnosis of NSCLC in testing set**

| **Marker** | **AUC** | **95% CI** | **Sensitivity** | **Specificity** | **Accuracy** |
| --- | --- | --- | --- | --- | --- |
| TP73-AS1 | 0.843 | 0.66-0.98 | 70% | 90% | 79% |
| CRNDE | 0.804 | 0.58-0.95 | 63% | 90% | 75% |
| CEA | 0.733 | 0.49-0.88 | 60% | 90% | 73% |
| CYFRA21-1 | 0.648 | 0.46-0.82 | 49% | 90% | 67% |
| TP73-AS1+CRNDE | 0.885 | 0.77-1.03 | 76% | 90% | 82% |
| TP73-AS1+CRNDE+CEA | 0.919 | 0.76-1.15 | 79% | 90% | 84% |
| TP73-AS1+CRNDE+CEA+CYFRA21-1 | 0.925 | 0.78-1.20 | 81% | 90% | 85% |

**Supplementary Table S7**

**Correlation between TP73-AS1 expression and clinicopathological characteristics of 212 patients with NSCLC**

**TP73-AS1 expression**

**Variable Low(%) High(%) P value**

| Age(years) | | | |
| --- | --- | --- | --- |
| <60 | 49(47) | 55(53) | 0.315 |
| ≥60 | 57(53) | 51(47) |  |
| Gender |  |  |  |
| Male | 50(51) | 48(49) | 0.406 |
| Female | 56(49) | 58(51) |  |
| Smoking status | | | |
| Nonsmoker | 54(48) | 59(52) | 0.323 |
| Smoker | 52(53) | 47(47) |  |
| Histology | | | |
| Adenocarcinoma | 47(47) | 53(53) | 0.368 |
| SCC | 59(53) | 52(47) |  |
| Tumor size (Cm) | | | |
| <3 | 62(62) | 38(38) | 0.014 |
| ≥3 | 44(39) | 68(61) |  |
| TNM stage | | | |
| Ⅰ-Ⅱ | 100(60) | 66(46) | 0.005 |
| ⅢA | 6(13) | 40(87) |  |
| Lymph node metastasis | | | |
| Yes | 99(60) | 67(40) | 0.007 |
| No | 7(15) | 39(85) |  |

**Supplementary Table S8**

**Correlation between CRNDE expression and clinicopathological characteristics of 212 patients with NSCLC**

**CRNDE expression**

**Variable Low(%) High(%) P value**

| Age(years) | | | |
| --- | --- | --- | --- |
| <60 | 47(45) | 57(55) | 0.242 |
| ≥60 | 59(55) | 49(45) |  |
| Gender |  |  |  |
| Male | 49(47) | 55(53) | 0315 |
| Female | 57(53) | 51(47) |  |
| Smoking status | | | |
| Nonsmoker | 56(48) | 61(52) | 0.319 |
| Smoker | 50(53) | 45(47) |  |
| Histology | | | |
| Adenocarcinoma | 45(45) | 56(55) | 0.337 |
| SCC | 59(53) | 52(47) |  |
| Tumor size (Cm) | | | |
| <3 | 60(62) | 37(38) | 0.018 |
| ≥3 | 46(40) | 69(60) |  |
| TNM stage | | | |
| Ⅰ-Ⅱ | 97(58) | 69(42) | 0.006 |
| ⅢA | 6(13) | 40(87) |  |
| Lymph node metastasis | | | |
| Yes | 101(61) | 65(39) | 0.003 |
| No | 5(11) | 41(89) |  |

**Supplementary Table S9**

**Univariate and multivariate analyses of factors for predicting disease free survival in patients with stage ⅠB - ⅢA NSCLC**

|  | **Univariate analysis** | |  | **Multivariate analysis** | |  |
| --- | --- | --- | --- | --- | --- | --- |
| **Variables** | **HR** | **95% CI** | ***P* value** | **HR** | **95% CI** | ***P* value** |
| Age (≥65y vs 65y) | 1.38 | 0.65-2.92 | 0.36 | － | － | － |
| Gender (male vs female) | 1.08 | 0.53-2.18 | 0.74 | － | － | － |
| Smoking status (smoker vs nonsmoker) | 1.59 | 0.68-3.87 | 0.22 | － | － | － |
| Histology (AD vs SCC) | 1.45 | 0.71-3.48 | 0.41 | － | － | － |
| Lymph node metastasis (positive vs negative) | 3.20 | 1.68-5.92 | 0.006 | 2.65 | 1.15-5.66 | 0.019 |
| TNM stage (Ⅰ-Ⅱ vs ⅢA ) | 3.42 | 1.84-6.23 | 0.002 | 2.79 | 1.21-5.83 | 0.015 |
| Plasma TP73-AS1 (high vs low) | 3.39 | 1.78-6.26 | <0.001 | 2.87 | 1.29-6.13 | 0.006 |
| Plasma CRNDE (high vs low) | 3.24 | 1.73-6.08 | <0.001 | 2.53 | 1.02-5.34 | 0.021 |
| CEA (≥ 5ng/ml vs ＜ 5 ng/ml) | 3.15 | 1.61-5.92 | 0.009 | 1.86 | 0.73-4.65 | 0.105 |
| CYFRA21-1 (≥ 5 ng/ml ≥ vs ＜ 5 ng/ml) | 2.74 | 0.96-5.37 | 0.015 | 1.55 | 0.62-4.18 | 0.342 |

DFS, disease free survival; NSCLC, non-small cell lung cancer; AD, adenocarcinoma; SCC, squamous cell carcinoma;

HR, hazard ratio; CI, confidence interval, CEA, carcinoembryonic antigen
